# Antagonizing effects of membrane-acting androgens on the eicosanoid receptor OXER1 in prostate cancer

**DOI:** 10.1038/srep44418

**Published:** 2017-03-14

**Authors:** Konstantina Kalyvianaki, Veronika Gebhart, Nikolaos Peroulis, Christina Panagiotopoulou, Fotini Kiagiadaki, Iosif Pediaditakis, Michalis Aivaliotis, Eleni Moustou, Maria Tzardi, George Notas, Elias Castanas, Marilena Kampa

**Affiliations:** 1 Laboratory of Experimental Endocrinology, School of Medicine, University of Crete, Heraklion, GR-71003, Greece; 2 Institute of Anatomy II, University Hospital Jena, Germany; 3 Department of Pharmacology, School of Medicine, University of Crete, Heraklion, GR-71003, Greece; 4 Institute of Molecular Biology & Biotechnology, Foundation of Research & Technology-Hellas (IMBB-FORTH), Heraklion, Greece; 5 Proteomics Facility at Institute of Molecular Biology and Biotechnology, Foundation of Research and Technology-Hellas, Heraklion, Crete; 6 Department of Pathology, School of Medicine, University of Crete, Heraklion, GR-71003, Greece.

## Abstract

Accumulating evidence during the last decades revealed that androgen can exert membrane initiated actions that involve signaling via specific kinases and the modulation of significant cellular processes, important for prostate cancer cell growth and metastasis. Results of the present work clearly show that androgens can specifically act at the membrane level via the GPCR oxoeicosanoid receptor 1 (OXER1) in prostate cancer cells. In fact, OXER1 expression parallels that of membrane androgen binding in prostate cancer cell lines and tumor specimens, while *in silico* docking simulation of OXER1 showed that testosterone could bind to OXER1 within the same grove as 5-OxoETE, the natural ligand of OXER1. Interestingly, testosterone antagonizes the effects of 5-oxoETE on specific signaling pathways and rapid effects such as actin cytoskeleton reorganization that ultimately can modulate cell migration and metastasis. These findings verify that membrane-acting androgens exert specific effects through an antagonistic interaction with OXER1. Additionally, this interaction between androgen and OXER1, which is an arachidonic acid metabolite receptor expressed in prostate cancer, provides a novel link between steroid and lipid actions and renders OXER1 as new player in the disease. These findings should be taken into account in the design of novel therapeutic approaches in prostate cancer.

Prostate cancer cells are highly dependent for their growth on testosterone (at least at the initial stages of the disease), with chemical castration by the administration of anti-androgen, being the primary line of treatment^1^. However, after a rather short time period (18–36 months) castration resistance develops and prostate cancer cells can grow independently of androgens. Hence, it seems (as recently shown by van der Sluis and his colleagues^2^) that, even at this stage, prostate cancer cells still dependent on hormones for migration, invasion and ultimately metastasis. Indeed, testosterone has been shown to induce migration and invasion of prostate cancer cells^2^ and serum testosterone levels to be correlated with a high-grade pathology and Gleason score^3^. These findings strongly designate testosterone as an important player in prostate cancer, with its mechanism of action requiring thorough investigation.

Androgen actions are classically mediated via intracellular androgen receptors (AR) that belong to the nuclear receptor superfamily. AR dimerizes and translocates to the nucleus after androgen binding, affecting gene expression. However, during the last fifteen years, a large amount of evidence points out an alternative mode of androgen action, that is initiated at the cell membrane, involves rapid signaling via specific kinases and modulates a significant number of cellular processes^4^. Previous work demonstrated that membrane androgen sites are present in a number of physiological (T lymphocytes, macrophages, spermocytes, sperm, osteoblasts)^5^,^6^,^7^, and cancer cells (prostate, breast, colon)^8^,^9^,^10^. In prostate and breast cancer cell lines, we have shown that membrane-acting androgens induce rapid cytoskeletal changes, resulting in the modulation of the adhesive and migratory capacity of the cells, as well as that they decrease cell growth and induce apoptosis^11^,^12^,^13^. Additionally, we have reported membrane-initiated specific genomic effects, different from those induced by intracellular AR activation^14^.

Based on these data it is now accepted that and rogens can exert membrane initiated actions, even though the nature of the receptor(s) involved has not been elucidated yet. A number of studies suggest the involvement of intracellular AR or a splice variant that may translocate to the membrane, via palmitoylation, similar to that occurring in ERα^15^, since AR also contains the required palmitoylation motif^15^. However, there are data that support the involvement of (an)other membrane protein(s). These include the inability of classical AR antagonists (flutamide, cyproterone acetate) to inhibit membrane initiated androgen actions^11^,^16^, the existence of rapid androgen actions in cells lacking classical AR^17^ and the inhibition of membrane-initiated androgen actions by pertussis toxin, indicating a GPCR involvement^18^. In fact, recent publications have identified two different GPCR proteins with characteristics of membrane androgen receptors: the G Protein-Coupled Receptor Family C Group 6 Member A^19^,^20^,^21^ and the zinc transporter protein, ZIP9^22^.

In the present work we characterized the GPCR oxoeicosanoid receptor 1 (OXER1), as a specific membrane receptor that mediates rapid effects of androgens in prostate cancer cells. We provide evidence that membrane acting testosterone can, in fact, antagonize the effects of 5-oxoETE, the endogenous ligand of OXER1, on modulating actin cytoskeleton, migration and specific initiated intracellular signaling, while we show that OXER1 expression and testosterone membrane binding coexist in prostate cancer tumor specimen.

## Results

### Affinity purification and characterization of membrane androgen binding sites

**Figure 1:**
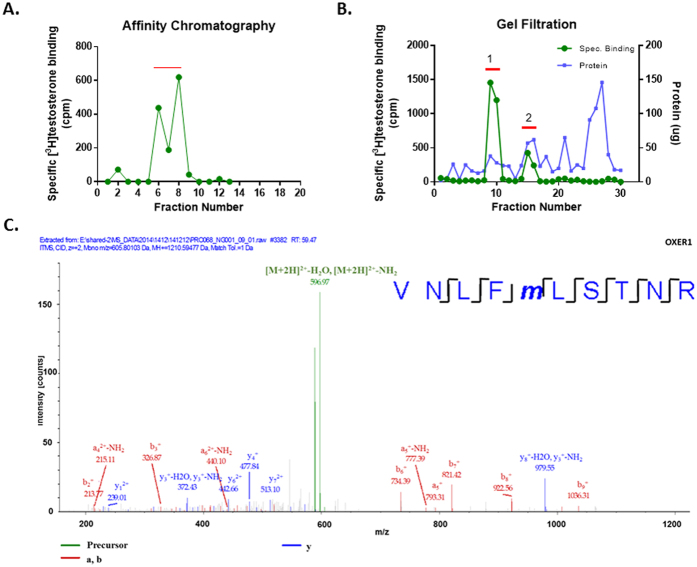
Specific [^3^H]-Testosterone binding in the eluates from the affinity chromatography column (**A**) and gel filtration (**B**); protein concentration in each fraction after gel filtration is also presented Representative graphs of four independent experiments are presented. (**C**) Mass spectrometric identification of OXER1 by one peptide. The MS/MS spectrum of the identified OXER1 peptide is shown where the identified peptide fragments are indicated in green, blue and red. The amino-acid sequence of the peptide is shown with the characteristic identified fragments.

Previously, we have identified specific membrane binding sites in prostate and breast cancer cell plasma membranes^12^,^13^,^16^,^23^, by [^3^H]Testosterone binding experiments and FACS, using a fluorescent impermeable testosterone analog (Testosterone-BSA-FITC). Here, we isolated plasma membranes from the prostate cancer cell line DU145, not expressing the classical intracellular AR. Isolated membranes were solubilized with CHAPS and loaded on an affinity chromatography column previously coupled to testosterone-BSA, through the covalent binding of BSA to the substrate. Proteins attached to testosterone were dissociated and eluted at a low pH and [^3^H]testosterone specific binding was assayed (Fig. 1A). Fractions 6, 7 and 8, exhibiting the highest specific binding were pooled and subjected to gel filtration chromatography. Eluates were equally tested for testosterone binding (Fig. 1B). Specific testosterone binding did not (as expected) correlate with the protein content of the fractions. Two major peaks (peak 1: fractions 9–10 and peak 2: fractions 15–16) were pooled separately and analyzed by mass spectrometry. A number of proteins were identified (Supplementary Table S1). First, it should be pointed out that neither the classical androgen receptor and any of its splice variants, nor the two recently proposed GPCR androgen receptors (GPRC6A and ZIP9)^19^,^20^,^21^,^22^ were identified in the filtration eluates. Among the identified proteins, only one, in the first peak, had characteristics matching those of a membrane receptor. This protein corresponds to the oxoeicosanoid receptor 1 (OXER1 or GPR170) with a molecular weight of 46 KD. Interestingly, a search in the evolutionary conserved regions database (http://ecrbrowser.dcode.org/) revealed that OXER1 is conserved in primates (100%), with a partial conservation (48%) in opossum, but it is absent in canines, rodents, birds and lower vertebrates, suggesting that this testosterone-OXER1 interaction is specific to primates (Supplementary Figure S1).

### Detection of OXER1 in prostate cancer cells lines and its interaction with steroids

**Figure 2:**
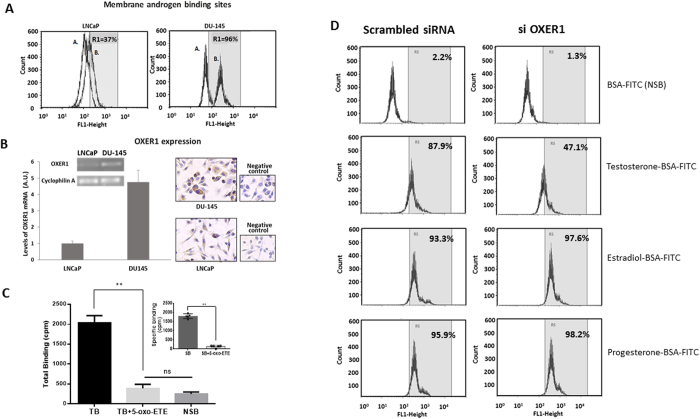
Membrane androgen binding sites on LNCaP and DU-145 cells assayed by flow cytometry. (**A**) Cells were labelled with either BSA-FITC (peak A) or Testosterone-BSA-FITC (peak B) and the percentage of cells that specifically bind testosterone-BSA-FITC is given by Gate R1. (**B**) OXER1 expression at the mRNA and protein level. OXER1 mRNA expression was assayed by Real Time PCR and was normalized to the expression of Cyclophilin A. Photos present LNCaP cells and DU-145 cells stained with an anti-OXER1 antibody. Representative graphs and photos of three independent experiments are presented. (**C**) Binding of [H^3^]testosterone (20 nM) on DU-145 isolated plasma cell membranes in the absence (Total Binding) or in the presence of 5-oxoETE (10^−6^ M). Non labelled testosterone (10^−5^ M) was utilized for non-specific binding (NSB). Insert shows specific binding. Data presented are the mean ± SEM of three independent experiments (**p < 0.001, NS: non-significant). (**D**) Membrane steroid binding sites on DU-145 cells transfected with a specific siRNA for OXER1 (right panel), or a scrambled siRNA (left panel) and assayed by flow cytometry. Cells were labelled with either BSA-FITC for non-specific binding (NSB) or testosterone-BSA-FITC, estradiol-BSA-FITC or progesterone-BSA-FITC for androgen, estrogen and progesterone binding sites respectively. The percentage of the specific steroid-BSA-FITC on cells is also shown (Gate R5). Figures are representative of three independent experiments.

We examined the expression of OXER1 both at mRNA and protein level in different cell lines of prostate (DU-145 and LNCaP) and breast cancer (T47D and SKBR3). OXER1 mRNA and protein were detected in all tested cell lines, with different levels of expression (Fig. 2A,B and Supplementary Table S2). Among them and in accordance to previous reports^24^, DU-145 prostate cancer cells exhibit the highest levels of OXER1. In fact, this variation in OXER1 expression totally correlates with membrane testosterone binding analyzed by FACS, as shown in Fig. 2A,B and Supplementary Table S2, further supporting OXER1 as a membrane androgen binding protein.

The endogenous ligand of OXER1 is the 5-oxo-eicosatetraenoic acid^25^,^26^. Here, in the absence of a labeled ligand for OXER1, we tested whether 5-oxo-ETE could displace binding of [^3^H]testosterone from plasma membranes. As presented in Fig. 2C, addition of 10^−6^ M 5-Oxo-ETE completely displaces radiolabeled testosterone, suggesting that these two compounds compete for binding to the same protein. In addition, membrane binding of testosterone-BSA-FITC was also assayed in DU-145 cells that were transfected with specific siRNA for OXER1. Testosterone-BSA-FITC binding was inhibited by 46% compared to cells that were transfected with a scrambled RNA. Additionally, this knocking down of OXER1 was specific for testosterone, as estradiol and progesterone membrane binding was not influenced by OXER1 knock-down (Fig. 2D).

**Figure 3:**
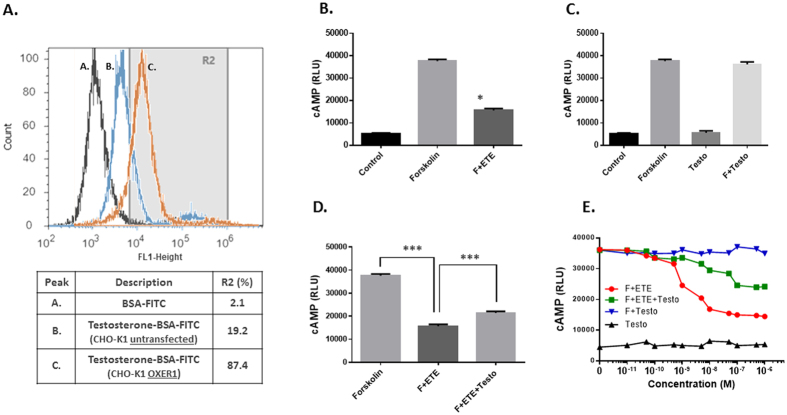
Membrane androgen binding sites on CHO-K1 cells overexpressing or not OXER1 as assayed by flow cytometry. Cells were labelled with either BSA-FITC (peak A) for non-specific binding or Testosterone-BSA FITC for specific binding (peaks B and C for untransfected and OXER1 overexpressing CHO-K1 cells respectively). The percentages of cells that specifically bind testosterone-BSA-FITC were obtained by Gate R2 and are presented in the table below the histograms. Figures are representative of three independent experiments. **B–D.** The effect of 5-oxoETE (10^−7^ M) and testosterone-BSA (10^−6^ M) on cAMP production by CHO-K1 cells overexpressing OXER1. Forskolin (F,15 μM) was utilized as a cAMP inducer. **E.** Dose response effect of 5-oxoETE and testosterone-BSA on cAMP production. Data are presented as a mean ± SEM of three independent experiments (*p < 0.01, *** p < 0.001).

In addition, we have tested the binding of Testosterone-BSA-FITC in CHO-K1 cells, not expressing OXER1 and the same cells stably transfected with the human OXER1 gene. Testosterone-BSA labels a small percentage (19.2%) of untransfected CHO-K1 cells. However, after transfection, 87.4% of cells express a specific membrane testosterone binding (Fig. 3A), further suggesting that OXER1 is a membrane androgen binding protein.

### Testosterone-BSA as an inhibitor of OXER1 signaling

One of the main molecular events reported for OXER1 is the inhibition of cAMP generation, due to its coupling to a G_αi_ protein^26^. Here, we verified this inhibitory effect, in CHO-K1 cells, stably transfected with the human OXER1 gene (Fig. 3B). The specific OXER1 agonist 5-oxo-ETE abolishes the forskolin stimulated cAMP production, with an apparent IC_50_ of ~2 × 10^−9^ M. Surprisingly, testosterone does not modify either the basal, or the forskolin stimulated cAMP production (Fig. 3C). In contrast, when cells were pre-incubated with testosterone and then provided with 5-oxo-ETE, the effect of the OXER1 agonist was reversed (~65%) by testosterone (at concentrations 10-times higher than 5-Oxo-ETE) (Fig. 3D and E). This unexpected finding suggests that testosterone (as well as its membrane-only acting analog testosterone-BSA) can act via OXER1 and antagonizes the effect of 5-OxoETE.

**Figure 4:**
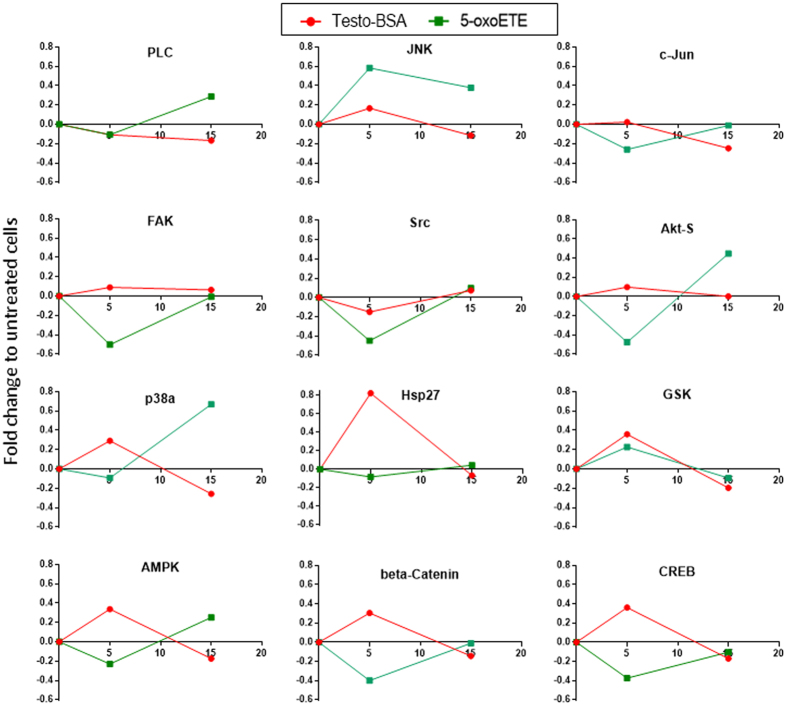
Changes to the phosphorylation of different kinases (assayed by a Human Phospho-Kinase Array Kit) under the influence of testosterone-BSA (10^−7^ M), or the OXER1 ligand, 5-OxoETE (10^−7^ M), for 5 and 15 min. Data are presented as a fold change compared to untreated cells (time = 0). Twelve different kinases were examined: PLC (Phospholipase C), JNK (c-Jun N terminal kinase), c-Jun, FAK (Focal Adhesion Kinase), c-src, Akt-s (Akt with a serine phosphorylation), p38α (Mitogen-activated Protein Kinase, MAPK), hsp27 (Heat Shock Protein 27), GSK3a/b (Glycogen Synthase kinase 3a/b), AMPK (5′ adenosine monophosphate-activated protein kinase), beta-Catenin, CREB (cAMP response element-binding protein). Blotted membranes are presented in Supplementary Figure S2.

Post-receptor signaling of OXER1, in addition to the inhibition of cAMP production includes G_ai_ and G_β/γ_ specific signaling cascades. They include Ca^2+^ mobilization, PI3K/Akt, PLC, JNK and ERK1/2 activation^27^,^28^. In addition, previous work^4^,^29^ established an intracellular rapid testosterone signaling, involving Ca^2+^ mobilization, FAK, PI3K/Akt, and p38 MAPK^30^. Here, using a kinase array, we have examined the effect of testosterone-BSA and 5-OxoETE, in DU-145 cells. We have restricted our analysis at 5 and 15 min, in order to assay the direct, rapid signaling effects of the agents (see membrane blots in Supplementary Figure S2). In Figure 4, the effect of 5-OxoETE and impermeable testosterone-BSA on 12 different significantly modified signaling molecules is presented. In the majority of cases (with the exception of src and GSK), the effect 5-OxoETE and testosterone -BSA are opposite, supporting an antagonistic effect of testosterone-BSA via OXER1. It is further to be noted that, as with all GPCRs, downstream signaling of OXER1 may be due to Gαi or Gβ/γ^28^. We have therefore, based on previous data, established a possible 5-OxoETE and testosterone-BSA signaling pathway, as we analyze in the Discussion section.

### Testosterone-BSA inhibits the rapid effect of 5-oxoETE on actin cytoskeleton and the migratory capacity of prostate cancer

**Figure 5:**
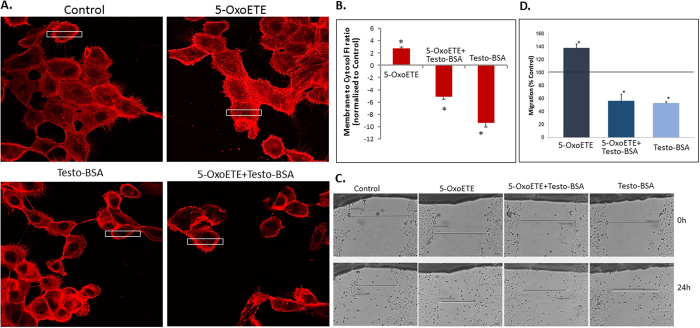
(**A**) Redistribution of actin filaments in DU-145 cells by 5-OxoETE (10^−7^ M), testosterone-BSA (10^−6^ M) and their combination. Cells were incubated for with the agents for 10 min and the redistribution of filamentous actin was determined with rhodamine-phalloidin staining and confocal laser microscopy. Magnification x400. Similar results were obtained in three independent experiments. (**B**) The intensity of actin staining at different cell compartments was derived by image analysis after sampling an area (white box in A) across a cell. Ten different random cells were assayed in each experimental condition and in three independent experiments. Results are plotted as a ratio of the fluorescence intensity (FI) at the membrane and the cytosol normalized to control (vehicle treated cells). Negative values indicate an actin shift to membrane compared to control (*P < 0.05 versus control). Horizontal line designates control. (**C**,**D**) The effect of 5-OxoETE (10^−7^ M), testosterone-BSA (10^−6^ M) and their combination was also tested on DU-145 cell migration in a wound healing assay. The healing progress of a linear scratch performed on a cell monolayer was photographed (time point 0 and 24 h). Representative photos are shown in (**C**) and the migration rate of each treatment compared to control (vehicle treated) cells is given in (**D**) Results (% of difference in migration over control cells) are the Mean ± SEM of three experiments performed in triplicate (*P < 0.05). Horizontal line designates control.

A rapid, phenotypic effect of OXER1 activation in WBC is the induction of chemotaxis and cell migration, due to a direct effect of actin polymerization^25^,^28^. On the other hand, actin polymerization, leading to a migration arrest has also been reported for membrane-acting testosterone^10^,^11^,^12^,^16^,^31^. These divergent results may be due to the differential cell distribution of the actin, induced by 5-Oxo-ETE, or testosterone. Indeed, in DU145 cells, highly expressing OXER1, 5-oxoETE triggers actin cytoskeleton rearrangements, with increased stress fiber formation and enhanced focal adhesions, compatible with an increased migratory capacity (Fig. 5A). In contrast, testosterone-BSA specifically redistributes actin towards the membrane, with the absence of stress fibers. The addition of both agents leads to a reversion of 5-oxo-ETE effect by testosterone-BSA. The opposing actin distribution in the cytoplasm and the sub-membrane space was further quantified in Fig. 5B.

Actin cytoskeleton rearrangements are directly implicated to cell migration, survival and apoptosis. Here we examined, for the first time, the effect of 5-oxo-ETE on prostate cell migration, and the implication of testosterone-BSA on this effect. Similarly, to the effect of the agent in neutrophils and lymphocytes^25^,^28^, OXER1 equally increases the migration of DU145 cells, (Fig. 5C,D), while testosterone-BSA opposes this effect. We have restricted our analysis at 24 hours, as testosterone-BSA is a very potent pro-apoptotic agent^12^ and prolonged incubation lead to an apoptotic effect, biasing the effects of migration assay.

### *In silico* molecular docking of testosterone on OXER1

**Figure 6:**
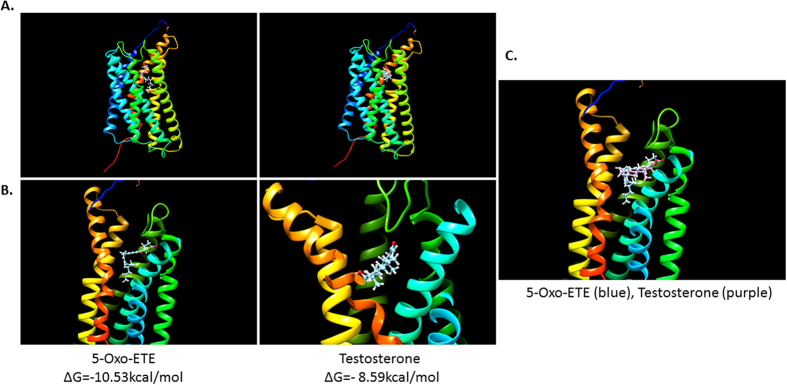
In silico molecular docking. Photos present a crystallographic model of OXER1 (with the most favorable QMEAN scoring using SWISS-MODEL) and the most probable (based on the calculated ΔGs values) docking simulations (performed on the SwissDock server and visualized by the UCSC Chimera program) for 5-OxoETE and testosterone-BSA alone (**A**). (**B**) represents a magnified picture. (**C**) Magnification of the binding groove with the superposition of both ligands.

An *in silico* docking simulation of OXER1 binding of 5-OxoETE and testosterone is presented in Fig. 6. Although we are aware of the limited validity of our approach, due to the absence of crystal structure of OXER1, our analysis provides some elements about the observed interaction of the two ligands. Indeed, the most favorable model shows that 5-OxoETE binds to a receptor grove, near the extracellular part of the receptor, being mainly associated with helices 3, 4 and 5 and the extracellular hinge of helices 4 and 5. The most energetically favorable solution for testosterone binding to OXER1 associates it within the same grove as for 5-OxoETE; however, in view of the smaller molecular radius of testosterone, a preferential interaction with chains 3 and 5 is observed. The superposing of the two ligands show that testosterone matches the binding elements of the carboxylic part of 5-OxoETE.

### Clinical evidence about testosterone-BSA-OXER1 interaction in prostate cancer specimens

The above data strongly suggest that testosterone and its membrane impermeable analog testosterone-BSA bind, in an antagonistic manner, on OXER1, in experimental settings of human prostate cancer cells. In this section, we try to establish further clinicopathological evidence about the expression and clinical significance of OXER1 in clinical specimens of prostate cancer.

At a first approach, using the cBioportal for cancer genomics (http://www.cbioportal.org/index.do)^32^,^33^, we have analyzed all TCGA data for OXER1 expression. As presented in Supplementary Figure S3A, a great disparity of expression is found, with higher expression in hepatocellular carcinoma, and the lowest in adrenocortical carcinoma. From the same resource, we have analyzed the expression of OXER1 in primary and metastatic prostate carcinoma (Supplementary Figure S3B and C respectively)^34^. Interestingly, the highest expression was found in hepatic and bone metastases and the lowest one in metastasis in the lymph nodes.

In contrast to metastatic prostate cancer, data from TCGA regarding primary prostate adenocarcinomas (Supplementary Figure S3B) showed a great disparity in OXER1 levels of expression. We therefore extracted gene expression and clinicopathological data from The Cancer Genome Atlas, restricting our analysis in patients’ samples, in which both normal and tumor tissue was analyzed. However, in most of these samples there is a significant discrepancy in the proportion of cancer epithelium/stroma/normal tissue (varying from 100/0/0 to 19/77/3 or 50/0/50) and a great disparity of lymphocyte (0–20%) and monocyte (0–40%) infiltration. Since OXER1 is highly expressed in different infiltrating cells^26^, we retain in our analysis only tumors that have at least 50% cancerous tissue and less that 15% lymphocyte/monocyte infiltrates. Analysis of the remaining 43 normal/tumor samples showed that OXER1 transcripts show significantly lower expression (p < 0.0001) in tumor than in normal prostate tissue (Supplementary Figure S3D) and show no relation steroid hormone (ESR1, AR, PGR) gene expression, or protein expression (ER, PR, AR), to the Gleason score of the tumor or the survival of patients (not shown). However, in this analysis, we have not further corrected for the epithelial-stroma content of the tumor.

**Figure 7:**
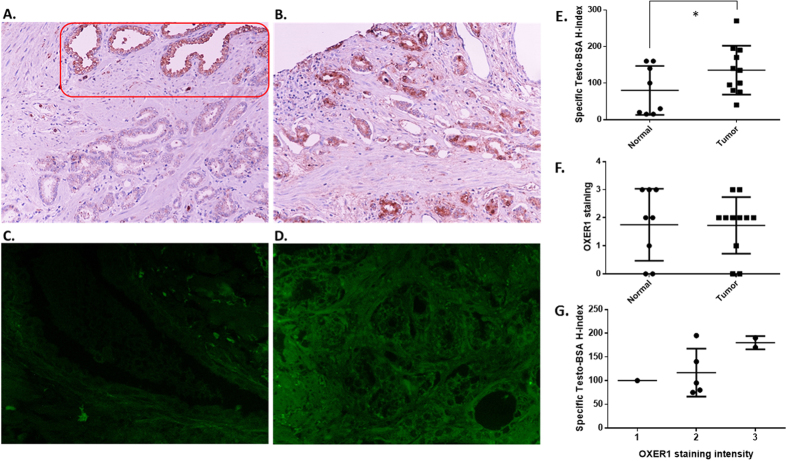
OXER1 and membrane androgen binding sites expression in prostate cancer cases. Representative pictures of low (**A**) and high (**B**) OXER1 expression and low (**C**) and high (**D**) membrane androgen binding sites expression (stained with testosterone-BSA-FITC). Red square indicates an area of normal tissue with high OXER1 expression. Comparison of expression levels of membrane androgen binding sites (**E**) and OXER1 (**F**) in normal and cancerous prostate tissue. **G.** Relation of membrane androgen binding sites and OXER1 in prostate cancer. Staining intensity was evaluated in a scale of 1–3, H-score: Histology score, see Material and Methods for calculation.

We have further constructed a tissue microarray with paired normal and tumor prostate tissue from a limited number of patients and stained it for OXER1 and membrane androgen binding sites, in order to provide a proof of principle of OXER1-Testosterone-BSA membrane binding (Fig. 7). In accordance with our previous findings^8^, testosterone-BSA membrane binding was increased in cancer, as compared to adjacent non-tumoral tissue (Fig. 7C,D and quantitation on E). In contrast, OXER1 immunoreactivity did not show a significant difference between cancerous and non-cancerous tissue (Fig. 7A,B and quantitation in F). In this respect, our data do not confirm the decreased cancer expression observed in mRNA analysis of cases in the TCGA data (Supplementary Figure S3D). This could be attributed to a possible difference of mRNA processing and mRNA stability in cancer tissue, a phenomenon also observed in other neoplasms, or the different degree of epithelial/tumor content of the analyzed specimens. Comparison of OXER1 and testosterone-BSA staining revealed a parallel increase of staining intensity between the two parameters in prostate cancer (Fig. 7G), further suggesting that OXER1 might be a membrane androgen binding protein.

## Discussion

OXER1 (5-oxo-6E,8Z,11Z,14Z-eicosatetraenoic acid receptor) is a GPCR that has been deorphanized almost 15 years ago^26^,^35^,^36^,^37^. It mediates the biological actions of 5-oxoeicosatetraenoic acid (5-oxoETE), a product of the metabolism of arachidonic acid by 5-lipoxygenase (5-LOX) and peroxidase. It is highly expressed in inflammatory cells, (eosinophils, neutrophils, lymphocytes and monocytes), in liver, kidney, spleen and lung tissue and in cancer cells, including prostate and breast^24^,^26^,^38^, as also presented here. It has been mainly studied in white blood cells, were it mediates the chemoattractant effect of 5-oxoETE^39^. In prostate cancer, OXER1 has a survival promoting effect^40^; siRNA for OXER1 significantly reduces the viability of prostate cancer cells and lately the use of agents that suppress 5-LOX activity or LOX-mediated signaling pathways have been proposed as new therapeutic tools in cancer^27^. Downstream signaling events related to OXER1 are inhibition of cyclic AMP, due to its coupling to Gα(_i/o_) protein, and an increased Ca^2+^ mobilization. Additionally, phosphorylation of PI3K, Akt and ERK1/2 have been found as the result of OXER1 activation, with the former being involved in the chemoattractant effect and the later in PLA_2_ activation^25^,^27^,^38^, while the involvement of PKCδ and ζ has been also described^41^.

Here, we present evidence that OXER1 is a membrane receptor for androgens since: (1) through affinity chromatography and MALDI-TOFF/MS analysis in purified membranes from DU145 prostate cancer cells we identified OXER1 as a testosterone-binding protein; (2) we verified OXER1 expression in both prostate and breast cancer cells that were previously used to study the existence of membrane testosterone binding sites and membrane initiated actions of testosterone^11^,^12^,^13^,^16^; (3) DU-145 cells expressed the highest mRNA levels of OXER1, a finding that is concordant with the high membrane androgen binding capacity of these cells; (4) knock-down of OXER1 expression by siRNA specifically reduced testosterone-BSA –FITC membrane binding, while induced overexpression of OXER1 led to significantly enhanced binding of impermeable testosterone; (5) Finally we report that OXER1 is present in prostate cancer tissues (our data from a TMA of a limited number of paired prostate cancer/normal specimens and data from The Human Protein Atlas (http://www.proteinatlas.org/) and that this expression parallels testosterone-BSA binding, suggesting that this system could be a potential therapeutic target in prostate cancer^42^,^43^. However, OXER1 protein expression in prostate cancer specimens did not match tumor mRNA content. In fact, a meticulous analysis of 43 paired tumor/normal prostate cancer cases from TCGA revealed that OXER1 transcripts are lower in tumor than in normal tissue, pointing out that epigenetic elements might also be implicated in the expression of OXER1 in prostate cancer.

**Figure 8:**
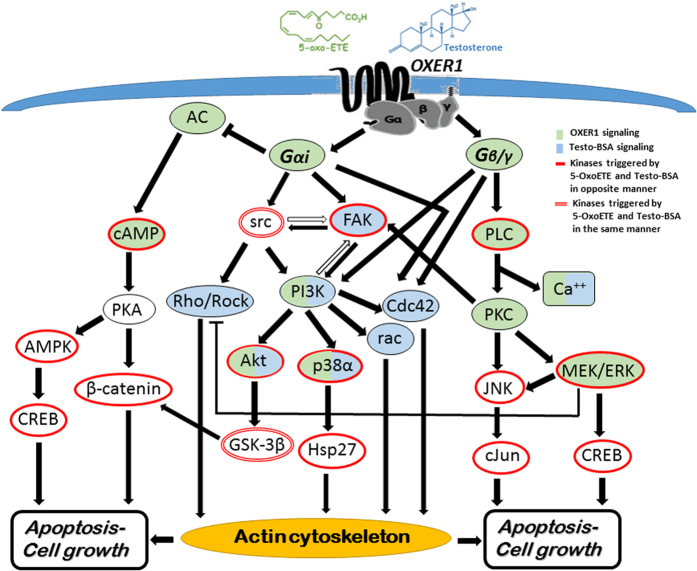
5-OxoETE and Testosterone-BSA proposed signaling via OXER1. The diagram includes kinases previously described to mediate 5-OxoETE via OXER1 effects (green fill) and testosterone effects (light blue fill) and also indicates the kinases found to be triggered in the present work (red outline). See the legend of Fig. 4 for the abbreviations used.

In order to further support our findings we studied in parallel the effect of testosterone-BSA and 5-oxoETE (the specific OXER1 ligand) on cell signaling cascades and actin cytoskeleton in DU-145 cells, based on our previous reports for specific membrane-initiated testosterone actions in these cells^29^,^30^. In addition, molecular docking studies show that both ligands might bind at the same grove of the receptor molecule, interacting with the same membrane loops, although testosterone mimics the binding of the carboxylic only part of 5-oxo-ETE. The major signaling pathways that we have previously described to be crucial for testosterone-BSA actions, such as FAK, PI3K, p38α, can indeed also be triggered by OXER1, but, surprisingly, testosterone-BSA and 5-oxoETE had opposing effects on these kinases. This suggests that testosterone does not act as an agonist on OXER1. This was further supported by the reversing effect of testosterone-BSA on 5-oxoETE inhibition of cAMP. Figure 8 summarizes the major signaling pathways that could be initiated by OXER1 and shows the kinases triggered by testosterone-BSA and 5-oxoETE. It includes findings of the present work and previous ones and clearly shows that indeed testosterone-BSA shares a similar (opposing) signaling with that of 5-oxoETE.

One of the major effects of membrane testosterone action is the rearrangement of actin cytoskeleton and an effect in the migratory capacity of the cells^11^,^12^,^16^. Similarly, 5-oxoETE has been shown to have a chemoattractant effect in white blood cells, by inducing actin polymerization and enhanced migration^26^,^27^,^28^. In DU-145 prostate cancer cells, 5-oxoETE rearranges actin cytoskeleton and induces migration, an effect that is inhibited in the presence of testosterone-BSA. This also greatly supports our initial findings of an antagonistic action of testosterone on OXER1.

All the above presented data strongly support that OXER1 is a membrane receptor that specifically can mediate the rapid effect of androgens. However, novel questions arise: (1) is OXER1 the only membrane androgen binding site? The plausible answer is no. In spite of the fact that we did not identify in our affinity purification any other putative membrane protein, we acknowledge the fact that two groups have identified two other proteins (ZIP9 and GPRC6A) related to the extranuclear action of androgen. However, the G Protein-Coupled Receptor Family C Group 6 Member A (GPRC6A)^19^,^20^,^21^ activation stimulated prostate cancer cell proliferation^44^, in contrast to our previous findings that membrane acting androgens induce prostate cancer cell apoptosis^12^,^13^; furthermore, GPRC6A is not specific for androgen binding. The zinc transporter protein, ZIP9^22^ was also found in prostate cells and tumors and seems to be specific for androgens. However, this protein is reported to induce cell apoptosis in a peculiar setting of serum-starved cells that is by itself a strong proapoptotic stimulus. Additionally, it is not clear whether ZIP9 is involved in other membrane initiated rapid androgen effects. Finally, zinc being a necessary factor for the correct conformation of the androgen receptor, it is not clear how the intracellular AR may be modified under the Zn-transporting effects of ZIP9. (2) What is the physiological role of OXER1 in prostate tissue and especially in prostate cancer? The physiological ligand of OXER1 (5-oxo-ETE) is produced by the 5-hyrdoxy eicosanoid dehydrogenase (5-HEDH), after the specific action of 5-LOX. 5-HEDH is found in different tissues, including the prostate^27^. However, it is still unclear how an exclusive intracellular metabolic intermediate like 5-oxo-ETE may interact with a membrane receptor. One possibility might be the passive diffusion of 5-oxo-ETE through the plasma membrane, as suggested by Sarveswaran and Ghosh^24^. Another possibility might be the 5-oxo-ETE release by apoptotic neutropils and dying tumor cells^27^. Here, we propose a third mechanism, namely the interaction of OXER1 with circulating androgen, eliciting rapid, membrane initiated, effects in tumor cells, regulating their fate. Moreover, we provide the first hints about a possible antagonism of this tumor-promoting receptor, in addition to other specific 5-LOX inhibitors^45^. Finally, the present work provides a link between lipid and steroids actions and could possibly provide additional evidence in order to elucidate the epidemiological findings of increased prostate cancer incidence or recurrence in hyperlipidemias as a result of overweight and obesity^46^,^47^,^48^. Interestingly enough, a very recent report^49^ identified the 12(S)-Hydroxyeicosatetraenoic acid (12-HETE) receptor (GPR31, 12-HETER) in prostate cancer tissue, further linking arachidonic acid metabolites as novel potent modulators of prostate growth and providing further elements for novel therapeutic approaches in this disease.

In conclusion, we report that OXER1 is a testosterone binding molecule that is critical for rapid membrane initiated androgen actions. Whether membrane acting androgen achieve their membrane related effects through OXER1 or they merely act as antagonists to the OXER1 natural ligand 5-oxoETE needs to be further elucidated. Further studies should also focus on the physiological relevance of this molecule in prostate cancer biology as it may be a potential novel therapeutic target for this disease.

## Materials and Methods

### Cell culture and materials

The DU-145, LNCaP, T47D and SKBR3 cell lines were purchased from DSMZ (Braunschweig, Germany), and were cultured in RPMI supplemented with 10% fetal bovine serum (FBS), at 37 °C, 5% CO_2_. All media were purchased from Invitrogen (Carlsbad, USA) and all chemicals from Sigma (St. Louis, MO), unless otherwise stated.

### Plasma membrane isolation

Cells cultured in a 225 cm^2^ flask (approximately 30 × 10^6^ cells) were scraped, using a cell scraper in plain PBS, then centrifuged at 1500 rpm for 5 min. Pelleted cells were used for the plasma membrane isolation using the “Qproteome Plasma Membrane Protein kit” (Qiagen), according to the manufacturer’s instructions. The kit utilizes a ligand specific for molecules on the plasma membrane and magnetic beads that bind to the ligand and ensures the specific isolation of plasma membranes. Isolated plasma membranes were solubilized by Tris-membrane buffer with CHAPS (50 mM Tris-HCL 0.1 mM CaCl_2_, 1 mM MgCl_2_, 5 mM KCl, 10 mM CHAPS pH 7.4) and centrifuged at 27000 rpm, in Beckman XL-90 Ultracentrifuge (Type 70 Ti Rotor), 4 °C for 1 h. The supernatant was collected and its protein concentration was measured using a spectrophotometer at 280 nm (NanoDrop 2000, Thermo Scientific, Cheshire, UK).

### Affinity chromatography

Solubilized membranes were added to an affinity chromatography column (1 ml Bed Volume HiTrap NHS-activated HP, GE Healthcare Life Sciences), which was covalently coupled with Testosterone 3-(O-carboxy-methyl)oxime:BSA, through the BSA moiety, according to the manufacturer’s instructions. Solubilized membranes were incubated overnight at 4 °C. The following day, the column was washed with 5–10 ml Tris-Membrane Buffer containing 0.1% Tween 20 and eluted using 3 ml of elution buffer (30 mM CH_3_COOH, pH 3.0). 13 eluate fractions were collected and protein concentration was measured using a spectrophotometer at 280 nm (NanoDrop 2000, Thermo Scientific, Cheshire, UK).

### Binding Studies

A binding assay, using radiolabeled testosterone, was performed on each eluate, in order to identify the fractions exhibiting a specific testosterone binding. For each fraction, Total Binding was assayed using a 50 μl eluate volume, additioned with 50 μl [^3^H] Testosterone (specific activity 95 Ci/mmol, Amersham-Pharmacia, Buckinghabshire, UK, 20 nM) and 100 μl of Assay Buffer (Tris-Membrane buffer + freshly added 1 mM PMSF and 1 μg/ml Aprotinin) and compared to non-specific binding (50 μl eluate, 50 μl [^3^H]Testosterone (20 nM), 50 μl DHT 4 × 10^−6^ M and 50 μl of Tris-membrane Buffer). Samples were incubated overnight on a shaker at 4 °C. The next day non-bound radiochemical was removed by Charcoal (500 μl 0.4% Charcoal, 0.04% Dextran T70 in assay buffer) for 10 min at 4 °C, followed by centrifugation at 4 °C for 20 mins at 2000 g. Four hundreds μl of the supernatant was added to 4 ml of scintillation cocktail and radioactivity was counted in a β-counter with a 60% efficiency for Tritium.

### Gel filtration

Eluates with a specific testosterone binding were pooled and applied on a Sephadex G-150 (Pharmacia) column (10 ml Bed Volume). The column was eluted with 15 ml Tris-Membrane buffer (50 mM Tris-HCl pH 7.4) and thirty eluates of approximately 0.5 ml were collected. Their protein concentration was measured at 280 nm (NanoDrop 2000, Thermo Scientific, Cheshire, UK) and their ability for specific testosterone binding was tested, as described above.

### Mass spectrometry-based identification of proteins

Fractions that exhibited specific testosterone binding were collected and subjected to mass spectrometry based proteomic analysis^50^. Briefly, after determination of protein content, fractions were dried in a speed-vacuum centrifuge and resuspended in the minimum required volume of 6 M urea, 2 M thiourea, 1% n-octylglucoside in 20 mM ammonium bicarbonate. The proteins were reduced and alkylated, digested in-solution for 3 h with endoproteinase Lys-C (1/100 w/w) (Waco, Bioproducts, Richmond, VA, USA), diluted four times with 20 mM ammonium bicarbonate and digested overnight with sequencing grade modified trypsin (1/100 w/w) (Promega Corp., Madison, WI, USA). Tryptic peptides were dried in a speed-vacuum centrifuge and dissolved in formic acid (5% in ultra-pure water, v/v). All samples were desalted on conditioned home-made pre-columns packed with C18 extraction disks (Empore^TM^ 3 M, MN, USA) and eluted stepwise with 80% Methanol and 5% Formic Acid. All elution fractions were collected, speed-vacuum centrifuged and diluted in 5% formic acid for further MS analysis.

Protein identification by nLC-ESI-MS/MS was done on a LTQ-Orbitrap XL coupled to an Easy nLC (Thermo Scientific, Cheshire, UK). The sample preparation and the LC separation was performed as described in ref. 51 with minor modifications. Briefly, the dried peptides were dissolved in 20 μl 0.5% formic acid, aqueous solution and the tryptic peptide mixtures were separated on a reversed-phase column (Reprosil Pur C18 AQ, particle size = 3 μm, pore size = 120 Å), fused silica emitters 100 mm long with a 75 μm internal diameter (Thermo Scientific, Cheshire, UK), packed in-house, using a pressurized (35 to 40 bars of helium) packing bomb (Loader kit SP035, Proxeon BioSystems, Denmark). The nLC flow rate was 200 nl min^−1^. Tryptic peptides were separated and eluted in a linear water-acetonitrile gradient and injected into the mass spectrometer as described in detail in^50^,^51^. MS survey scans were acquired in the Orbitrap from 200 to 2,000 m/z at a resolution of 60,000 and for the MS/MS, precursor isolation at 1.6 m/z was performed by the quadrupole (Q). Fragmentation of twenty most intense ions by collision induced dissociation (CID) with normalized collision energy of 35% and rapid scan MS analysis were carried out in the ion trap. The dynamic exclusion duration was set to 15 s with 10 ppm tolerance around the selected precursor and its isotopes. The AGC target values were set to 4.0 × 10^5^ and 1.0 × 10^4^ and maximum injection times were 50 ms and 35 ms for MS and MSn scans, respectively. MS raw data were analyzed in Proteome Discoverer 1.4.0 (Thermo Fischer Scientific, Waltham, MA USA) using Mascot 2.3.01 (Matrix Science, Ltd. London, UK) search algorithm. Spectra were run against the Human theoretical proteome (Last modified July 9, 2014. Version 153), containing 140,330 entries^52^ and a list of common contaminants^53^. Search parameters employed are described in detail in ref. 54. Final peptide and protein lists were compiled in Scaffold (version 4.4.1.1, Proteome Software; Portland, OR) employing criteria as previously described^54^.

### Detection of membrane steroid binding sites by flow cytometry

Cultured cells were detached by scraping, washed once with PBS and suspended in PBS at a density of 10^6^ cells/ml. Then, cells were incubated for 10 min with either testosterone-BSA-FITC (Testosterone3-(O-carboxymethyl) oxime—BSA-FITC) (10^−6^ M) or BSA-FITC (10^−6^ M) for assaying membrane androgen binding sites and non-specific binding respectively. FITC labelled estradiol-BSA (estradiol3-(O-carboxymethyl)oxime-BSA-FITC) and progesterone-BSA (progesterone 3-(O-carboxymethyl) oxime-BSA-FITC) were also utilized at a similar concentration in order to detect estrogen and progesterone membrane binding sites. Before each experiment, stock solutions of BSA conjugates were mixed with dextran (0.05 mg/ml) and charcoal (50 mg/ml) for 30 min (DCC treatment) in order to remove any potential contamination with free steroid, centrifuged at 3000 g for 10 min and passed through a 0.22 mm filter for removing any remaining solid material. Cells were analyzed by flow cytometry using the Attune Acoustic Focusing Cytometer (Thermo Fischer Scientific, Waltham, MA USA), in a sample size of 20,000 cells gated on the basis of forward and side scatter.

### OXER1 siRNA silencing

DU-145 cells were seeded at an initial of 300000 cells/well in a 6 well plate with 2 ml medium per well. Specific siRNAs for OXER1 (AM16708, ID: 212835) and scrambled siRNAs (ID:149158, both from Thermo Fischer Scientific, Waltham, MA USA) were transfected using Lipofectamine 2000^®^ (Thermo Fischer Scientific, Waltham, MA USA) according to standard protocols. After 24 h, fresh medium was added and 24 hours later cells were detached and used for the detection of membrane steroid binding sites by flow cytometry as described above.

### Real Time PCR

Total cell RNA was isolated using Total RNA isolation Kit (NucleoSpin RNA II, NucleoSpin RNA L, Macherey-Nagel) following the manufacturer’s instructions and subjected to RT-PCR. cDNA was synthesized using the PrimeScript^TM^ 1st strand cDNA Synthesis Kit by Takara Bio Inc. A mixture of extracted RNA with 1 μl of random 6mers (50 μΜ), 2 μl of dNTP mixture (10 mm each) and RNase-free DH20, in a total volume of 10 μl, was incubated at 65 °C for 5 min and then cooled immediately on ice. Afterwards 4 μl of 5 X PrimeScript^TM^ Buffer, 0.5 μl (20 units) of RNase Inhibitor (40 U/μl), 1 μl (200 units) of PrimeScript^TM^ RTase (200 U/μl) and 4.5 μl of RNase free dH_2_O were added for further incubation at 30 °C for 10 minutes and then at 42 °C for 60 minutes. The enzymes were inactivated by incubation at 70 °C for 5 minutes and then cooled at 4 °C. Generated cDNA was used to perform the amplification in a 10 μl reaction buffer, containing 2.5 μl of cDNA, 5 μl of master mix (KAPA SYBR FAST qPCR Master Mix, Kapa Biosystems, Inc. Wilmington, MA, USA), 1.5 μl of dH_2_O, 0.5 μl of the 3′-primer and 0.5 μl of the 5′-primer. For amplification of OXER1, PCR reactions were performed in an Applied Biosystems Step-One Plus^®^ apparatus, using the following primer pairs (synthesized by Eurofins Genomics, Ebersberg, Germany): 5′: AGG AGC CTT CCT TTT CCA GA and 3′: CGA CGA GAG CTC CTA CCA AC (amplicon size:119 bp). The number of cycles used was optimized to fall within the linear range of PCR amplification curve. Changes were normalized according to cyclophilin A expression (5′: ATG GTC AAC CCC ACC GTG T and 3′: TTC TGC TGT CTT TGG AAC TTT GTC). Reaction products were also resolved on 1.5% agarose gel containing 3 μl of ethidium bromide to determine the molecular sizes of the OXER1 amplicons. The gel images were acquired with BIO-RAD Molecular Imager ChemiDoc^TM^ XRS + .

### Kinase detection assay

Rapid changes to the phosphorylation of cellular kinases, under the influence of testosterone-BSA, or the OXER1 ligand, 5-OxoETE (5-Ketoeicosa-6E,8Z,11Z,14E-tetraenoic acid, Enzo Life Sciences) were determined by the use of a Human Phospho-Kinase Array Kit (Proteome Profiler^TM^ Array, R&D Systems, Minneapolis, USA) that assays 43 different kinases. In fact it utilizes specific antibodies for 43 kinases and 2 related total proteins captured on a nitrocellulose membrane, and the use of phospho-specific antibodies followed by chemiluminescent detection. DU145 cells were cultured in 6-well plates and treated for 5 or 15 min with testosterone-BSA, or 5-OxoETE (at a final concentration of 10^−6^ M, in order to obtain a maximal response). Then, cells were lysed with 1 ml lysis buffer per 10^7^ cells and after a 30 min incubation at 4 °C and centrifugation (14 000 × g, 5 min, 4 °C), 600 μg of protein lysate were applied onto the nitrocellulose membranes with the different captured antibodies in duplicates. All subsequent steps were carried according to the manufacturer instructions. Chemiluminescence was read in a BIO-RAD Molecular Imager ChemiDoc^TM^ XRS+, for 30 s until 10 min. Spot intensities were then quantified using the FIJI software (http://fiji.sc/), based on NIH Image J2. The mean values of duplicate spots were normalized with the average of the positive control spots and data are presented as a fold change compared to untreated cells (time = 0).

### cAMP assay

The effect of 5-oxo-ETE, testosterone –BSA and their combination, via OXER1, on cAMP levels was examined by the the cAMP Hunter™ eXpress OXER1 CHO-K1 GPCR Assay (DiscoverX, Fremont CA, USA) which utilizes CHO-K1 cells, stably overexpressing OXER1 and a gain-of-signal competitive immunoassay based on Enzyme Fragment Complementation (EFC) technology. The assay was performed according to manufacturer’s instructions and since OXER1 is a G_αi_-coupled receptor, forskolin (15 μM) was used in order to reveal the inhibitory effect of the molecule. Briefly, after an overnight incubation, CHO-K1 cells, seeded in 96-well plate (~30000 cells/well), were treated with serial dilutions (10^−6^–10^−11^ M) of the agents for 30 min at 37 °C. In order to reveal the 5-oxo-ETE antagonistic effect of testosterone, cells were pre-treated with the impermeable testosterone-BSA (activated charcoal purified, as described above) for 15 min at 37 °C. cAMP standard serial dilutions (2.31 × 10^−6^–3.91 × 10^−11^ M) were run in parallel as a standard curve, in the absence of cells. Subsequently, an immunocompetition reaction was induced by 1-hour incubation (at room temperature in the dark) with cellular cAMP, enzyme donor(ED) cAMP and anti-cAMP, followed by unbound ED-cAMP detection. Unbound ED-cAMP is free to complement with an Enzyme Acceptor in a three-hour incubation (at room temperature in the dark) to form active enzyme, which subsequently produces a luminescent signal. The amount of signal produced was read in a Microplate Fluorescence Reader (BIO-TEK Instruments Inc. Winooski, Vermont, USA) and is directly proportional to the amount of cAMP in the cells.

### Actin cytoskeleton visualization

DU-145 cells, growing on 8-well chamber slides, were treated with testosterone–BSA or 5-OxoETE (10^−6^ M), for 15 min. Subsequently, they were fixed in 4% paraformaldehyde for 10 min and permeabilized by 0.5% TritonX100 for 10 min. They were then incubated with 2% BSA for 15 min, followed by rhodamine-labeled phalloidine staining, for 45 min at room temperature. After two washes with PBS, slides were mounted with VECTASHIELD^®^ mounting medium with DAPI (Vector laboratories, Burlingame, CA, USA) and visualized using a confocal laser scanning module attached to a microscope equipped with an argon–krypton ion laser (CLSM, Leica TCS-NT).

### Cell migration assay

DU145 cells were cultivated in 12-well plates. Confluent cell monolayers were treated with mitomycin C (10 mg/ml) for 2 h, in order to inhibit cell proliferation. Afterwards, the medium with mitomycin C was removed and two straight lines were scratched in the monolayers, per well, with a p200 pipette tip to create cell-free area. Then, cells were treated with testosterone–BSA or 5-OxoETE, at a final concentration of 10^−6^ M. After the substances were added, the first images of the scratches under an inverted phase-contrast microscope (Vert.A1 ZEISS International, Jena, Germany) were acquired (time = 0). Another series of images were obtained after 24 h of incubation at 37 °C in order to assay the rate of colonization of the denuded area. The size of the denuded area was measured at time = 0 and at time = 24 h and normalized differences in migration (as % of control, non-treated cells) were estimated by subtracting the size of the denuded area from its size on time 0 and by dividing by size on t = 0. Three different points were measured per scratch-line. All conditions were assayed in triplicates.

### *In silico* molecular docking studies

OXER1 has not yet been crystalized. Therefore, simulation experiments were performed in the Swiss Model Biospace^55^, hosted in the University of Basel (http://swissmodel.expasy.org/interactive). Modeling is based on evolutionary information modeling of proteins, using already crystalized models^56^, after performing a BLAST and HHBlits^57^ against the primary amino acid sequence of structures contained in the Swiss Model template database (SMTL, last update: 2016-04-27). A total of 184 templates were found (see Supplementary Table S3 for a complete list of the retrieved sequences). The global and per-residue model quality has been assessed using the QMEAN scoring function^58^. For improved performance, weights of the individual QMEAN terms have been trained specifically for SWISS-MODEL. Three models were retained, using the P2Y purine receptor as a template (71% coverage identity). Results were exported in pdb files. The first model (presenting the most favorable QMEAN scoring) was retained for docking simulations. Ligand pdb and sdf files were produced by translating their SMILES notation to 2D coordinates by the use of the online translator at https://cactus.nci.nih.gov/translate.

Docking experiments were performed on the SwissDock server (http://www.swissdock.ch), based on the EADock DSS engine^59^,^60^. Visualizations were made by the use of the UCSC Chimera program, v1.11^61^.

### Immunocytochemistry

Immunocytochemistry for OXER1 was performed using a rabbit polyclonal antihuman OXER1 antibody (raised against an 18 amino acid peptide from 1st extracellular domain of human OXER1, Thermo Scientific, Cheshire, UK, Cheshire, UK) at an optimal dilution of 1:100 for a 45 min incubation, at room temperature. Counterstaining was performed using Harris’s hematoxylin (BIOSTAIN, Manchester, UK). The UltraVision LP Detection System: HRP Polymer Quanto (Thermo Scientific, Cheshire, UK, Cheshire, UK) was utilized, with Diaminobenzidine (DAB) as the chromogen for detection. Negative controls (omission of the primary antibody) were used in each run.

#### Prostate cancer specimen selection

Twelve prostate cancer specimens were selected from the Pathology Department archives of the University Hospital of Heraklion. Clinicopathological data have been retrieved from the database of the Department of Pathology. They are presented in Supplementary Table S4. This study was approved by the Research and Ethics Committee of the Heraklion University Hospital (1016/2016). All methods were performed in accordance with the relevant guidelines and regulations. As this study was retrospective, performed on surgical specimens, after the establishment of diagnosis and the application of appropriate treatment to patients and that this exploratory analysis would not derive on novel therapeutic modalities to involved patients, an individual informed consent was not considered necessary by the Ethics Committees. All patient records/information were anonymized and de-identified prior to analysis.

#### TMA construction

Representative areas were selected from tumor specimens by an expert pathologist, with the exclusion of necrotic areas. Depending on availability, representative areas from paired non tumoral prostate tissue from the same patients were selected. Three cores (0.4 mm) from each tumor and non tumoral blocks were retrieved by TMA Master (3DHISTECH Kft., Hungary) to cover reproducibility and tumor heterogeneity. From each TMA, 3 μm serial sections were serially cut for Hematoxylin-Eosin (HE) staining, mAR and OXER1 staining.

#### Membrane androgen binding sites and OXER1 staining

**Membrane androgen binding sites** were detected by the use of testosterone-BSA-FITC (4 molecules FITC and 8–12 molecules steroid/molecule BSA) or BSA-FITC (12 molecules FITC/molecule of BSA) for nonspecific binding which was systematically substracted. Slide sections were subjected to an overnight incubation at 40 °C for a mild melting of the embedding medium and dewaxed and rehydrated by a 2 h incubation at 37 °C in citrate buffer (0.01 mol/L, pH 6.2), and a wash in TBS (10 mmol/L, NaCl 150 mmol/L, pH 7.4). To minimize nonspecific absorption of albumin to membrane structures, all slides were pre-incubated with 3% BSA for 40 min. Then, the slides were washed in TBS and incubated for 1 h in the dark with either testosterone-BSA-FITC or BSA-FITC (10^−6^ M final concentration) in a buffer containing cyproterone acetate (10^−5^ M final concentration) in order to eliminate binding of the steroid conjugate to intracellular AR^8^. Afterwards they were rinsed with TBS and coversliped using VECTASHIELD^®^ mounting medium with DAPI (Vector laboratories, Burlingame, CA, USA) and examined in a fluorescence microscope (Olympus), Photographs were taken from each slide (at least five different fields) under 40x magnification and under identical conditions of exposure and fluorescence intensity.

For **immunohistochemical staining of OXER1**, slides were deparaffinized and subsequently hydrated by three cycles (5 min) of citrate buffer (0.01 M, pH 6.0) in a steamer, and treated with 3% hydrogen peroxide for 15 min. Then they were incubated with the anti OXER1 antibody (working dilution 1:50) (Thermo Scientific, Cheshire, UK, Cheshire, UK). Counterstaining was performed using Harris hematoxylin (BIOSTAIN, Manchester, UK). For the detection of tissue antibody binding, UltraVision LP Detection System: HRP Polymer Quanto (Thermo Scientific, Cheshire, UK, Cheshire, UK) was used, with Diaminobenzidine (DAB) as chromogen. Normal serum in place of the primary antibody was used in every run as a negative control. Slides were observed and photographed under 40x magnification in optical microscope (Olympus).

In all images the Histology score (H-score)^62^ was calculated, evaluating the intensity (in a scale of 1–3) and the percentage of positive staining, by the formula (%*1 + %*2 + %*3), ranging from 0–300. Total immunopositivity is expressed as a percentage of total positive cells (0–100), with membrane staining independently of the intensity of staining.

### Statistical Analysis

Analysis was performed by SPSS V21 (SPSS/IBM Inc, Chicago, USA) and Prism V6 (GraphPad Software Inc. San Diego CA) as detailed in the results. (p < 0.05 was retained as a significance threshold). Other specific statistical methods for gene and microarray analysis are described in the results section.

## Additional Information

**How to cite this article**: Kalyvianaki, K. *et al*. Antagonizing effects of membrane-acting androgens on the eicosanoid receptor OXER1 in prostate cancer. *Sci. Rep.* **7**, 44418; doi: 10.1038/srep44418 (2017).

**Publisher's note:** Springer Nature remains neutral with regard to jurisdictional claims in published maps and institutional affiliations.

## Supplementary Material

Supplementary Information
