# Supplementary material for: Antagonizing effects of membrane-acting androgens on the eicosanoid receptor OXER1 in prostate cancer
Source: Sci Rep. 2017 Mar 14;7:44418. doi: 10.1038/srep44418 (PMC5349529; doi:10.1038/srep44418)
Supplement: Supplementary Information [file srep44418-s1.pdf]

Antagonizing effects of membrane-acting androgens on the eicosanoid receptor OXER1 in prostate cancer

Konstantina Kalyvianaki, Veronika Gebhart, Nikolaos Peroulis, Christina Panagiotopoulou, Fotini Kiagiadaki, Iosif Pediaditakis, Michalis Aivaliotis, Eleni Moustou, Maria Tzardi, George Notas, Elias Castanas, Marilena Kampa

Synteny of the **Human[hg19] chr2:42989639-42991401** region

Dog [canFam2]

#	Relative position	Region	Coverage	Length	Genomic position	Homology interval	Alignment / TFBS

Fugu [fr3]

#	Relative position	Region	Coverage	Length	Genomic position	Homology interval	Alignment / TFBS

Chicken [galGal3]

#	Relative position	Region	Coverage	Length	Genomic position	Homology interval	Alignment / TFBS

Mouse [mm10]

#	Relative position	Region	Coverage	Length	Genomic position	Homology interval	Alignment / TFBS

Opossum [monDom5]

	#	Relative position	Region	Coverage	Length	Genomic position	Homology interval	Alignment / TFBS
☐	1	573-1426		48.4%	854	chr2:42990211-42991064	**chr1**:656560530-656561389 [860 bps]	Mulan

Rhesus macaque [rheMac2]

	#	Relative position	Region	Coverage	Length	Genomic position	Homology interval	Alignment / TFBS
☐	1	1-1763		100.0%	1763	chr2:42989639-42991401	**chr13**:42881201-42882964 [1.7 kb]	Mulan

Frog [xenTro3]

#	Relative position	Region	Coverage	Length	Genomic position	Homology interval	Alignment / TFBS

SUPPLEMENTARY FIGURE S1

Human OXER1 gene and its orthologs among different species.

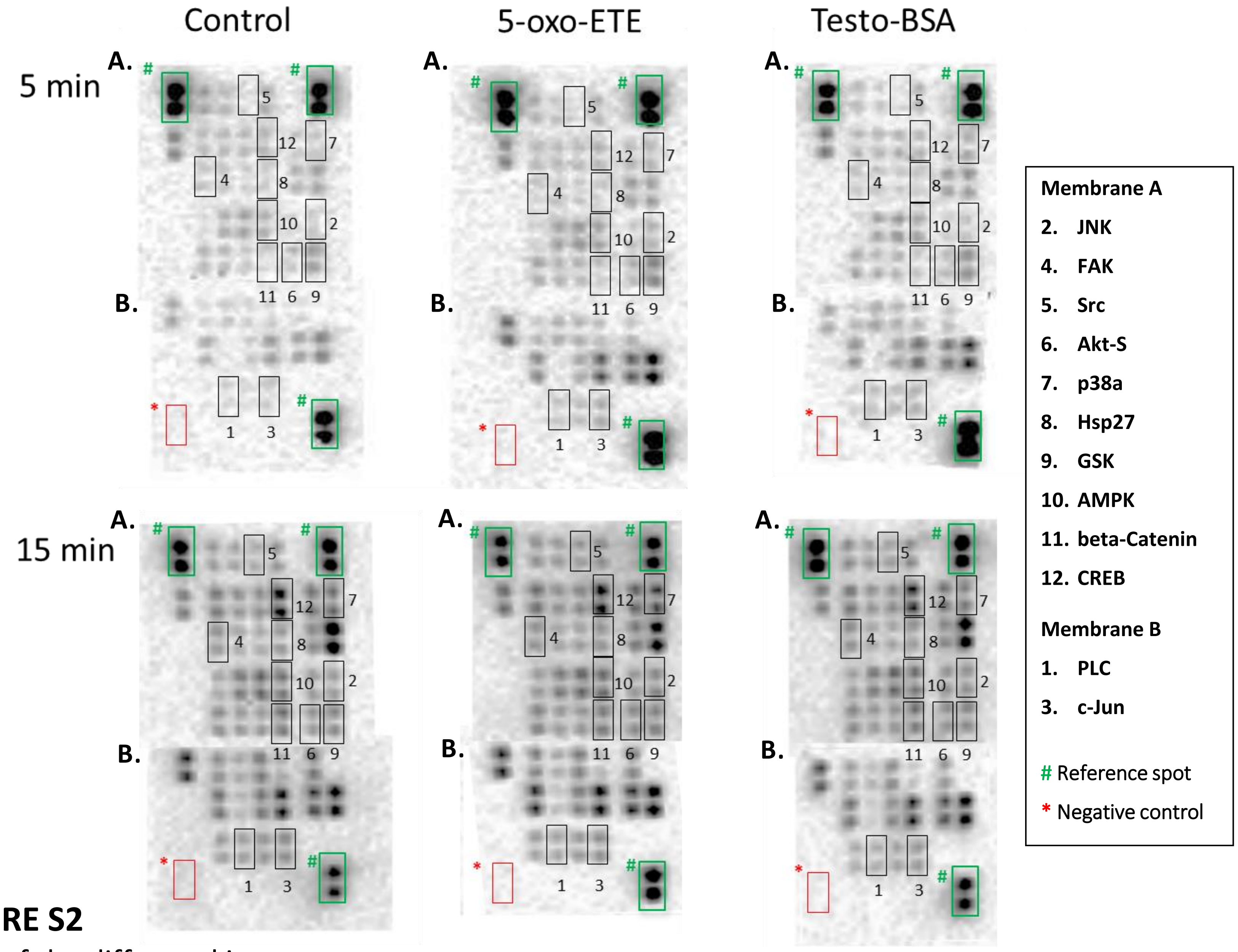

SUPPLEMENTARY FIGURE S2

Western blot membranes of the different kinases.

For each condition and time points, two blot membranes (A and B) for different kinases were used. Kinases significantly modified are shown in black boxes.

A.

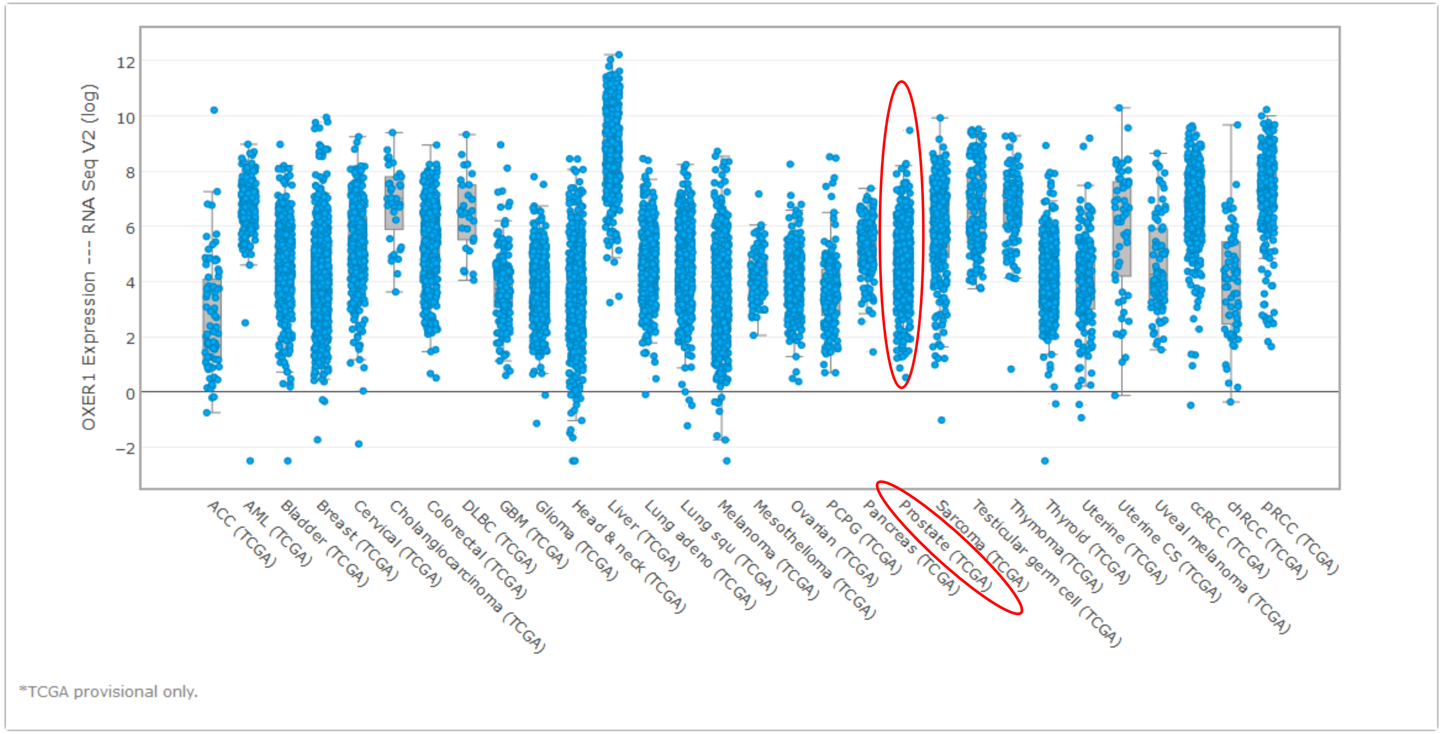

D.

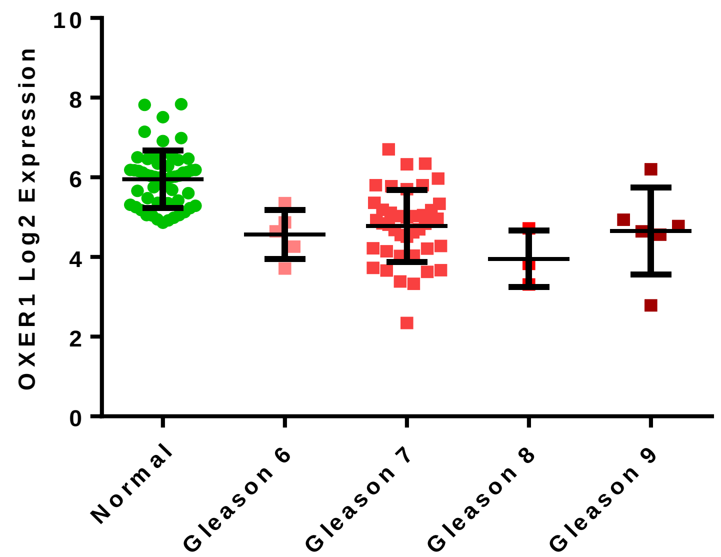

B.

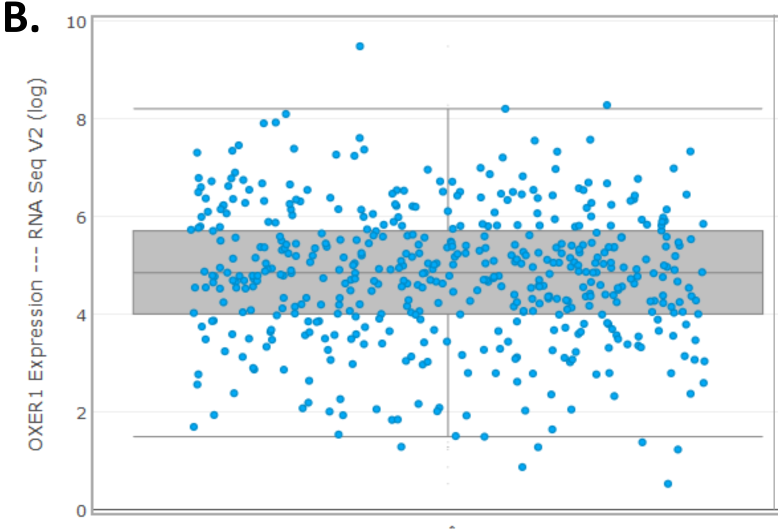

C.

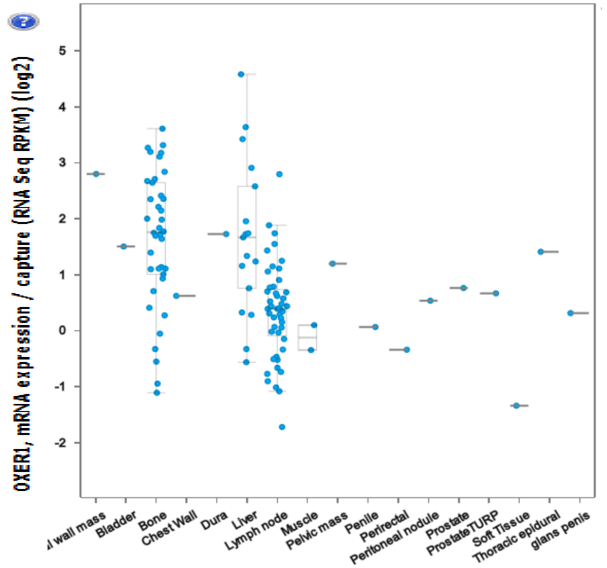

SUPPLEMENTARY FIGURE S3

OXER1 gene expression in different cancers.

(**A.**) Analysis of TCGA data for OXER1 expression using the cBioportal for cancer genomics including prostate cancer. Expression levels of OXER1 gene in different prostate cancer cases, primary (**B.**) and metastatic (**C.**). **(D.)** Expression levels of OXER1 gene in a subset of prostate cancer cases (normal tissue available, > 50% tumor parenchyma, <15% infiltration, see Results for more details) in correlation to normal tissue and Gleason Score.

SUPPLEMENTARY TABLE 1

Identified proteins by MS/ MS

MS/MS View:Identified Proteins	Accession Number	Molecular Weight	Peak 1	Peak 2
Keratin, type II cytoskeletal 1 OS=Homo sapiens GN=KRT1 PE=1 SV=6	K2C1_HUMAN	66 kDa	1	
Keratin, type I cytoskeletal 10 OS=Homo sapiens GN=KRT10 PE=1 SV=6	K1C10_HUMAN	59 kDa	1	
KH domain-containing, RNA-binding, signal transduction-associated protein 3 (Fragment) OS=Homo sapiens GN=KHDRBS3 PE=4 SV=1	H0YAQ3_HUMAN	21 kDa		0.55
C2ORF3 variant 2 OS=Homo sapiens GN=C2orf3 PE=2 SV=1	A4UHQ9_HUMAN	28 kDa		0.66
Leucine--tRNA ligase, cytoplasmic OS=Homo sapiens GN=LARS PE=2 SV=1	B4DER1_HUMAN	131 kDa		0.73
Serine/threonine-protein kinase WNK1 OS=Homo sapiens GN=WNK1 PE=4 SV=1	F5GWT4_HUMAN	225 kDa		0.58
Immunoglobulin superfamily DCC subclass member 4 OS=Homo sapiens GN=IGDCC4 PE=1 SV=1	IGDC4_HUMAN	134 kDa	0.95	
Septin-2 (Fragment) OS=Homo sapiens GN=SEPT2 PE=4 SV=1	H7C1T1_HUMAN	3 kDa	0.76	
Cytochrome P450 3A4 OS=Homo sapiens GN=CYP3A4 PE=1 SV=4	CP3A4_HUMAN	57 kDa		0.8
Isoform 2 of Myosin-11 OS=Homo sapiens GN=MYH11	MYH11_HUMAN	228 kDa		0.97
Isoform 2 of Protein arginine N-methyltransferase 8 OS=Homo sapiens GN=PRMT8	ANM8_HUMAN	44 kDa	0.78	
Glucose-fructose oxidoreductase domain-containing protein 1 OS=Homo sapiens GN=GFOD1 PE=1 SV=1	GFOD1_HUMAN	43 kDa	0.95	
Oxoeicosanoid receptor 1 OS=Homo sapiens GN=OXER1 PE=2 SV=1	OXER1_HUMAN	46 kDa	0.95	
Pre-mRNA cleavage complex 2 protein Pcf11 OS=Homo sapiens GN=PCF11 PE=1 SV=3	PCF11_HUMAN	173 kDa		0.97
Keratin, type II cytoskeletal 2 epidermal OS=Homo sapiens GN=KRT2 PE=1 SV=2	K22E_HUMAN	65 kDa	0.95	
Lymphocyte antigen 86 OS=Homo sapiens GN=LY86 PE=1 SV=1	LY86_HUMAN	18 kDa	0.95	
Bile acid receptor (Fragment) OS=Homo sapiens GN=NR1H4 PE=4 SV=1	H0YHD5_HUMAN	4 kDa	0.95	
Keratin, type II cytoskeletal 6A OS=Homo sapiens GN=KRT6A PE=1 SV=3	K2C6A_HUMAN	60 kDa	0.95	
Ubiquitin carboxyl-terminal hydrolase OS=Homo sapiens GN=USP7 PE=2 SV=1	B7Z815_HUMAN	126 kDa	0.98	
Zinc finger protein 518B OS=Homo sapiens GN=ZNF518B PE=2 SV=2	Z518B_HUMAN	120 kDa		0.97
Doublecortin domain-containing protein 1 OS=Homo sapiens GN=DCDC1 PE=4 SV=1	M0R2J8_HUMAN	201 kDa		0.97
Kelch repeat and BTB domain-containing protein 8 OS=Homo sapiens GN=KBTBD8 PE=2 SV=2	KBTB8_HUMAN	69 kDa		0.97
Protein kinase C OS=Homo sapiens GN=PRKCG PE=3 SV=1	F5H5C4_HUMAN	80 kDa		0.96
Hsc70-interacting protein (Fragment) OS=Homo sapiens GN=ST13 PE=4 SV=1	H7C3I1_HUMAN	16 kDa		0.96
Probable ATP-dependent RNA helicase DHX34 OS=Homo sapiens GN=DHX34 PE=1 SV=2	DHX34_HUMAN	128 kDa		0.96

SUPPLEMENTARY TABLE 2

OXER1 expression and androgen membrane binding sites in prostate and breast cancer cell lines. (A.U.: Arbitary Units)

Cell line	OXER1 Expression		Testosterone-BSA-FITC Binding
	mRNA (A.U.)	Protein	
**DU-145**	4.7	++++	86%
**LNCaP**	1.0	++	37%
**T47D**	1.1	++	35%
**SKBR3**	1.8	+++	75%

SUPPLEMENTARY TABLE 3: Retained models for the OXER1 3D modeling

Template	Seq Identity	Oligo-state	Found by	Method	Resolution	eq Similarit	Coverage	Description
4xnv.1.A	23.75	monomer	HHblits	X-ray	2.20Å	0.33	0.71	P2Y purinoceptor 1, Rubredoxin, P2Y purinoceptor 1
4xnw.1.A	23.75	monomer	HHblits	X-ray	2.70Å	0.33	0.71	P2Y purinoceptor 1,Rubredoxin,P2Y purinoceptor 1
4xnw.2.A	23.75	monomer	HHblits	X-ray	2.70Å	0.33	0.71	P2Y purinoceptor 1,Rubredoxin,P2Y purinoceptor 1
4ej4.1.A	25.52	monomer	HHblits	X-ray	3.40Å	0.32	0.68	Delta-type opioid receptor, Lysozyme chimera
3vw7.1.A	23.51	monomer	HHblits	X-ray	2.20Å	0.32	0.68	Proteinase-activated receptor 1, Lysozyme
4ntj.1.A	18.03	monomer	HHblits	X-ray	2.62Å	0.3	0.7	P2Y purinoceptor 12, Soluble cytochrome b562
4pxz.1.A	18.03	monomer	HHblits	X-ray	2.50Å	0.3	0.7	P2Y purinoceptor 12, Soluble cytochrome b562
4py0.1.A	18.03	monomer	HHblits	X-ray	3.10Å	0.3	0.7	P2Y purinoceptor 12, Soluble cytochrome b562
4djh.1.B	23.67	homo-dimer	HHblits	X-ray	2.90Å	0.32	0.67	Kappa-type opioid receptor, Lysozyme
4djh.1.A	23.67	homo-dimer	HHblits	X-ray	2.90Å	0.32	0.67	Kappa-type opioid receptor, Lysozyme
3oe8.1.B	20.96	homo-dimer	HHblits	X-ray	3.10Å	0.3	0.69	C-X-C chemokine receptor type 4, Lysozyme Chimera
4yay.1.A	21.75	monomer	HHblits	X-ray	2.90Å	0.31	0.68	Soluble cytochrome b562,Type-1 angiotensin II receptor
3oe8.2.A	20.83	homo-trimer	HHblits	X-ray	3.10Å	0.3	0.69	C-X-C chemokine receptor type 4, Lysozyme Chimera
3odu.1.A	20.83	homo-dimer	HHblits	X-ray	2.50Å	0.3	0.69	C-X-C chemokine receptor type 4, Lysozyme Chimera
3odu.1.B	20.83	homo-dimer	HHblits	X-ray	2.50Å	0.3	0.69	C-X-C chemokine receptor type 4, Lysozyme Chimera
3oe8.1.A	20.83	homo-dimer	HHblits	X-ray	3.10Å	0.3	0.69	C-X-C chemokine receptor type 4, Lysozyme Chimera
2ks9.1.A	22.7	hetero-oligome	HHblits	NMR	NA	0.32	0.67	Substance-P receptor
3oe0.1.A	20.49	hetero-oligome	HHblits	X-ray	2.90Å	0.3	0.69	C-X-C chemokine receptor type 4, Lysozyme Chimera
3oe9.1.B	20.49	homo-dimer	HHblits	X-ray	3.10Å	0.3	0.69	C-X-C chemokine receptor type 4, Lysozyme Chimera
3oe9.1.A	20.49	homo-dimer	HHblits	X-ray	3.10Å	0.3	0.69	C-X-C chemokine receptor type 4, Lysozyme Chimera
4rwd.1.A	24.91	monomer	HHblits	X-ray	2.70Å	0.32	0.67	Chimera protein of Human Delta-type opioid receptor and Escherichia coli Soluble cytochrome b562
4rwa.1.A	24.91	monomer	HHblits	X-ray	3.28Å	0.32	0.67	Chimera protein of Human Delta-type opioid receptor and Escherichia coli Soluble cytochrome b562
4grv.1.A	19.86	monomer	HHblits	X-ray	2.80Å	0.3	0.68	Neurotensin receptor type 1, lysozyme chimera
4n6h.1.A	25	monomer	HHblits	X-ray	1.80Å	0.32	0.67	Soluble cytochrome b562, Delta-type opioid receptor chimeric protein
4zud.1.A	21.35	monomer	HHblits	X-ray	2.80Å	0.31	0.67	Chimera protein of Soluble cytochrome b562 and Type-1 angiotensin II receptor
4ea3.1.A	23.13	homo-dimer	HHblits	X-ray	3.01Å	0.31	0.67	Fusion protein of Nociceptin receptor and cytochrome b562
5dhh.1.A	23.21	monomer	HHblits	X-ray	3.00Å	0.31	0.67	GPCR-BRIL Chimera
5dhh.2.A	23.21	monomer	HHblits	X-ray	3.00Å	0.31	0.67	GPCR-BRIL Chimera
4xes.1.A	20.42	monomer	HHblits	X-ray	2.60Å	0.3	0.68	Neurotensin receptor type 1, Endolysin chimera
4bv0.2.A	20.42	hetero-oligome	HHblits	X-ray	3.10Å	0.3	0.68	NEUROTENSIN RECEPTOR TYPE 1
4bv0.1.A	20.42	hetero-oligome	HHblits	X-ray	3.10Å	0.3	0.68	NEUROTENSIN RECEPTOR TYPE 1
4xee.1.A	20.85	monomer	HHblits	X-ray	2.90Å	0.3	0.67	Neurotensin receptor type 1, Endolysin chimera
4dkl.1.A	22.86	homo-dimer	HHblits	X-ray	2.80Å	0.31	0.67	Mu-type opioid receptor, lysozyme chimera
4bwb.2.A	20.49	hetero-oligome	HHblits	X-ray	3.57Å	0.3	0.67	NEUROTENSIN RECEPTOR TYPE 1
4bwb.1.A	20.49	hetero-oligome	HHblits	X-ray	3.57Å	0.3	0.67	NEUROTENSIN RECEPTOR TYPE 1
4zj8.1.A	22.78	monomer	HHblits	X-ray	2.75Å	0.31	0.67	human OX1R fusion protein to P.abysii glycogen synthase
3zev.1.A	19.86	monomer	HHblits	X-ray	3.00Å	0.3	0.67	NEUROTENSIN RECEPTOR 1 TM86V
4buo.1.A	19.86	hetero-oligome	HHblits	X-ray	2.75Å	0.3	0.67	NEUROTENSIN RECEPTOR TYPE 1
4buo.2.A	19.86	hetero-oligome	HHblits	X-ray	2.75Å	0.3	0.67	NEUROTENSIN RECEPTOR TYPE 1
4mbs.1.A	18.79	monomer	HHblits	X-ray	2.71Å	0.3	0.67	Chimera protein of C-C chemokine receptor type 5 and Rubredoxin
2lnl.1.A	24.19	monomer	HHblits	NMR	NA	0.32	0.66	C-X-C chemokine receptor type 1
3oe6.1.A	20.57	homo-dimer	HHblits	X-ray	3.20Å	0.3	0.67	C-X-C chemokine receptor type 4, Lysozyme Chimera
2x72.1.A	19.57	hetero-oligome	HHblits	X-ray	3.00Å	0.3	0.67	RHODOPSIN
4rws.1.A	20.64	hetero-oligome	HHblits	X-ray	3.10Å	0.3	0.67	C-X-C chemokine receptor type 4/Endolysin chimeric protein
4bvn.1.A	21.66	monomer	HHblits	X-ray	2.10Å	0.31	0.66	BETA-1 ADRENERGIC RECEPTOR
2ycw.1.A	21.74	monomer	HHblits	X-ray	3.00Å	0.31	0.66	BETA-1 ADRENERGIC RECEPTOR
2ycy.1.A	21.74	monomer	HHblits	X-ray	3.15Å	0.31	0.66	BETA-1 ADRENERGIC RECEPTOR
4gpo.1.A	21.74	homo-dimer	HHblits	X-ray	3.50Å	0.31	0.66	Beta-1 adrenergic receptor
5f8u.1.A	21.74	monomer	HHblits	X-ray	3.35Å	0.31	0.66	Beta-1 adrenergic receptor
2y03.1.A	21.74	monomer	HHblits	X-ray	2.85Å	0.31	0.66	BETA-1 ADRENERGIC RECEPTOR
2ycx.1.A	21.74	monomer	HHblits	X-ray	3.25Å	0.31	0.66	BETA-1 ADRENERGIC RECEPTOR

2ycz.1.A	21.74	monomer	HHblits	X-ray	3.65Å	0.31	0.66	BETA-1 ADRENERGIC RECEPTOR
3kj6.1.A	20.14	hetero-oligome	HHblits	X-ray	3.40Å	0.31	0.66	Beta-2 adrenergic receptor
2j4y.1.A	19.22	homo-dimer	HHblits	X-ray	3.40Å	0.3	0.67	RHODOPSIN
5a8e.1.A	21.74	monomer	HHblits	X-ray	2.40Å	0.31	0.66	BETA1 ADRENERGIC RECEPTOR
2rh1.1.A	19.78	monomer	HHblits	X-ray	2.40Å	0.3	0.66	beta-2-adrenergic receptor/T4-lysozyme chimera
3p0g.1.A	19.78	hetero-oligome	HHblits	X-ray	3.50Å	0.3	0.66	Beta-2 adrenergic receptor, Lysozyme
3sn6.1.D	20.29	hetero-oligome	HHblits	X-ray	3.20Å	0.31	0.66	Lysozyme, Beta-2 adrenergic receptor
2r4r.1.A	20.22	hetero-oligome	HHblits	X-ray	3.40Å	0.31	0.66	Beta-2 adrenergic receptor
3oax.1.A	19.29	monomer	HHblits	X-ray	2.60Å	0.3	0.67	Rhodopsin
1gzm.1.A	19.29	homo-dimer	HHblits	X-ray	2.65Å	0.3	0.67	RHODOPSIN
2i36.1.A	19.29	monomer	HHblits	X-ray	4.10Å	0.3	0.67	Rhodopsin
2i37.1.A	19.29	monomer	HHblits	X-ray	4.15Å	0.3	0.67	Rhodopsin
1l9h.3.B	19.29	homo-dimer	HHblits	X-ray	2.60Å	0.3	0.67	rhodopsin
4bey.1.A	18.86	hetero-oligome	HHblits	X-ray	2.90Å	0.29	0.67	RHODOPSIN
3c9m.1.A	18.86	monomer	HHblits	X-ray	3.40Å	0.29	0.67	Rhodopsin
4a4m.1.A	18.86	hetero-oligome	HHblits	X-ray	3.30Å	0.29	0.67	RHODOPSIN
3pxo.1.A	19.29	homo-dimer	HHblits	X-ray	3.00Å	0.3	0.67	Rhodopsin
1f88.1.B	19.29	homo-dimer	HHblits	X-ray	2.80Å	0.3	0.67	RHODOPSIN
1ln6.1.A	19.29	monomer	HHblits	NMR	NA	0.3	0.67	RHODOPSIN
1f88.1.A	19.29	homo-dimer	HHblits	X-ray	2.80Å	0.3	0.67	RHODOPSIN
3c9l.1.A	19.29	monomer	HHblits	X-ray	2.65Å	0.3	0.67	Rhodopsin
3cap.1.A	19.29	homo-dimer	HHblits	X-ray	2.90Å	0.3	0.67	Rhodopsin
1jfp.1.A	19.29	monomer	HHblits	NMR	NA	0.3	0.67	rhodopsin
4qkx.1.A	20.29	hetero-oligome	HHblits	X-ray	3.30Å	0.31	0.66	Beta-2 adrenergic receptor
5dsg.2.A	20.65	monomer	HHblits	X-ray	2.60Å	0.31	0.66	Muscarinic acetylcholine receptor M4,Endolysin,Endolysin,Muscarinic acetylcholine receptor M4
5dsg.1.A	20.65	monomer	HHblits	X-ray	2.60Å	0.31	0.66	Muscarinic acetylcholine receptor M4,Endolysin,Endolysin,Muscarinic acetylcholine receptor M4
2r4s.1.A	20.29	hetero-oligome	HHblits	X-ray	3.40Å	0.31	0.66	Beta-2 adrenergic receptor
4lde.1.A	20.36	hetero-oligome	HHblits	X-ray	2.79Å	0.31	0.65	Lysozyme, Beta-2 adrenergic receptor
4zwj.1.A	19	monomer	HHblits	X-ray	3.30Å	0.3	0.66	Chimera protein of human Rhodopsin, mouse S-arrestin, and T4 Endolysin
4gbr.1.A	20.73	hetero-oligome	HHblits	X-ray	3.99Å	0.31	0.65	Beta-2 adrenergic receptor
5cxv.1.A	23.36	monomer	HHblits	X-ray	2.70Å	0.31	0.65	Muscarinic acetylcholine receptor M1,Endolysin,Muscarinic acetylcholine receptor M1
4iaq.1.A	21.17	homo-dimer	HHblits	X-ray	2.80Å	0.31	0.65	Chimera protein of human 5-hydroxytryptamine receptor 1B and E. Coli soluble cytochrome b562
5c1m.1.A	22.88	hetero-oligome	HHblits	X-ray	2.10Å	0.31	0.65	Mu-type opioid receptor
4iar.1.A	21.17	monomer	HHblits	X-ray	2.70Å	0.31	0.65	Chimera protein of human 5-hydroxytryptamine receptor 1B and E. Coli soluble cytochrome b562
4u16.2.A	19.71	monomer	HHblits	X-ray	3.70Å	0.31	0.65	Muscarinic acetylcholine receptor M3,Lysozyme,Muscarinic acetylcholine receptor M3
4u16.1.A	19.71	monomer	HHblits	X-ray	3.70Å	0.31	0.65	Muscarinic acetylcholine receptor M3,Lysozyme,Muscarinic acetylcholine receptor M3
3ny8.1.A	19.27	monomer	HHblits	X-ray	2.84Å	0.3	0.65	Beta-2 adrenergic receptor, Lysozyme
2ziy.1.A	18.55	monomer	HHblits	X-ray	3.70Å	0.3	0.65	Rhodopsin
3ayn.1.A	18.55	monomer	HHblits	X-ray	2.70Å	0.3	0.65	Rhodopsin
4eiy.1.A	19.42	monomer	HHblits	X-ray	1.80Å	0.29	0.66	Adenosine receptor A2a/Soluble cytochrome b562 chimera
4ww3.1.A	18.61	monomer	HHblits	X-ray	2.80Å	0.3	0.65	Rhodopsin
4daj.1.A	19.78	monomer	HHblits	X-ray	3.40Å	0.31	0.65	Muscarinic acetylcholine receptor M3, Lysozyme
4daj.2.A	19.78	monomer	HHblits	X-ray	3.40Å	0.31	0.65	Muscarinic acetylcholine receptor M3, Lysozyme
4u14.1.A	19.78	monomer	HHblits	X-ray	3.57Å	0.31	0.65	Muscarinic acetylcholine receptor M3,Endolysin,Muscarinic acetylcholine receptor M3
3uzc.1.A	19.13	monomer	HHblits	X-ray	3.34Å	0.29	0.66	Adenosine A2A Receptor
4xt1.1.A	22.71	hetero-oligome	HHblits	X-ray	2.89Å	0.3	0.65	G-protein coupled receptor homolog US28
4xt3.1.A	22.71	hetero-oligome	HHblits	X-ray	3.80Å	0.3	0.65	G-protein coupled receptor homolog US28
4nc3.1.A	18.05	monomer	HHblits	X-ray	2.80Å	0.29	0.66	Chimera protein of human 5-hydroxytryptamine receptor 2B and E. Coli soluble cytochrome b562
4ib4.1.A	18.05	monomer	HHblits	X-ray	2.70Å	0.29	0.66	Chimera protein of human 5-hydroxytryptamine receptor 2B and E. Coli soluble cytochrome b562
3vga.1.A	19.57	hetero-oligome	HHblits	X-ray	3.10Å	0.29	0.66	Adenosine receptor A2a
3vg9.1.A	19.57	hetero-oligome	HHblits	X-ray	2.70Å	0.29	0.66	Adenosine receptor A2a
3pbl.2.A	21.85	monomer	HHblits	X-ray	2.89Å	0.31	0.64	D(3) dopamine receptor, Lysozyme chimera
3pbl.1.A	21.85	monomer	HHblits	X-ray	2.89Å	0.31	0.64	D(3) dopamine receptor, Lysozyme chimera

4mqs.1.A	18.25	hetero-oligome	HHblits	X-ray	3.50Å	0.3	0.65	Muscarinic acetylcholine receptor M2
3uon.1.A	18.25	monomer	HHblits	X-ray	3.00Å	0.3	0.65	Human M2 muscarinic acetylcholine, receptor T4 lysozyme fusion protein
3qak.1.A	19.34	monomer	HHblits	X-ray	2.71Å	0.3	0.65	Adenosine receptor A2a,lysozyme chimera
3eml.1.A	19.34	monomer	HHblits	X-ray	2.60Å	0.3	0.65	Human Adenosine A2A receptor/T4 lysozyme chimera
3rze.1.A	22.06	monomer	HHblits	X-ray	3.10Å	0.3	0.65	Histamine H1 receptor, Lysozyme chimera
4uhr.1.A	18.91	monomer	HHblits	X-ray	2.60Å	0.29	0.65	THERMOSTABILISED HUMAN A2A RECEPTOR
4ug2.1.A	18.91	monomer	HHblits	X-ray	2.60Å	0.29	0.65	THERMOSTABILISED HUMAN A2A RECEPTOR
2ydv.1.A	18.91	monomer	HHblits	X-ray	2.60Å	0.29	0.65	ADENOSINE RECEPTOR A2A
3pds.1.A	20.07	monomer	HHblits	X-ray	3.50Å	0.3	0.64	Fusion protein Beta-2 adrenergic receptor/Lysozyme
4phu.1.A	21.77	monomer	HHblits	X-ray	2.33Å	0.28	0.65	Free fatty acid receptor 1,Lysozyme
4z34.1.A	16.1	monomer	HHblits	X-ray	3.00Å	0.29	0.64	Lysophosphatidic acid receptor 1, Soluble cytochrome b562
4z35.1.A	16.1	monomer	HHblits	X-ray	2.90Å	0.29	0.64	Lysophosphatidic acid receptor 1,Soluble cytochrome b562
4z36.1.A	16.1	monomer	HHblits	X-ray	2.90Å	0.29	0.64	Lysophosphatidic acid receptor 1,Soluble cytochrome b562
3v2w.1.A	16.79	monomer	HHblits	X-ray	3.35Å	0.28	0.64	Sphingosine 1-phosphate receptor 1, Lysozyme chimera
4l6r.1.A	15.63	monomer	HHblits	X-ray	3.30Å	0.26	0.61	Soluble cytochrome b562 and Glucagon receptor chimera
4s0v.1.A	23.4	monomer	HHblits	X-ray	2.50Å	0.31	0.45	Human Orexin receptor type 2 fusion protein to P. abysii Glycogen Synthase
5ee7.1.A	15.75	monomer	HHblits	X-ray	2.50Å	0.26	0.35	Glucagon receptor,Endolysin,Glucagon receptor
4xnv.1.A	36.67	monomer	BLAST	X-ray	2.20Å	0.39	0.29	P2Y purinoceptor 1, Rubredoxin, P2Y purinoceptor 1
4xnw.1.A	36.67	monomer	BLAST	X-ray	2.70Å	0.39	0.29	P2Y purinoceptor 1,Rubredoxin,P2Y purinoceptor 1
4xnw.2.A	36.67	monomer	BLAST	X-ray	2.70Å	0.39	0.29	P2Y purinoceptor 1,Rubredoxin,P2Y purinoceptor 1
4djh.1.A	30.83	homo-dimer	BLAST	X-ray	2.90Å	0.37	0.29	Kappa-type opioid receptor, Lysozyme
4djh.1.B	30.83	homo-dimer	BLAST	X-ray	2.90Å	0.37	0.29	Kappa-type opioid receptor, Lysozyme
4n6h.1.A	33.33	monomer	BLAST	X-ray	1.80Å	0.36	0.28	Soluble cytochrome b562, Delta-type opioid receptor chimeric protein
4ej4.1.A	33.33	monomer	BLAST	X-ray	3.40Å	0.36	0.28	Delta-type opioid receptor, Lysozyme chimera
4jkv.1.B	13.18	homo-dimer	HHblits	X-ray	2.45Å	0.25	0.31	Soluble cytochrome b562, Smoothened homolog
4jkv.1.A	13.18	homo-dimer	HHblits	X-ray	2.45Å	0.25	0.31	Soluble cytochrome b562, Smoothened homolog
4n4w.1.A	13.18	homo-dimer	HHblits	X-ray	2.80Å	0.25	0.31	Chimera protein of smoothened receptor_BRIL and Cytochrome b(562)
3vw7.1.A	29.73	monomer	BLAST	X-ray	2.20Å	0.36	0.26	Proteinase-activated receptor 1, Lysozyme
4mbs.1.A	25.89	monomer	BLAST	X-ray	2.71Å	0.35	0.27	Chimera protein of C-C chemokine receptor type 5 and Rubredoxin
2lnl.1.A	32.43	monomer	BLAST	NMR	NA	0.36	0.26	C-X-C chemokine receptor type 1
5dsg.2.A	25.86	monomer	BLAST	X-ray	2.60Å	0.32	0.28	Muscarinic acetylcholine receptor M4,Endolysin,Endolysin,Muscarinic acetylcholine receptor M4
5dsg.1.A	25.86	monomer	BLAST	X-ray	2.60Å	0.32	0.28	Muscarinic acetylcholine receptor M4,Endolysin,Endolysin,Muscarinic acetylcholine receptor M4
4dkl.1.A	30.91	homo-dimer	BLAST	X-ray	2.80Å	0.37	0.26	Mu-type opioid receptor, lysozyme chimera
5c1m.1.A	30.91	hetero-oligome	BLAST	X-ray	2.10Å	0.37	0.26	Mu-type opioid receptor
4rws.1.A	30.63	hetero-oligome	BLAST	X-ray	3.10Å	0.35	0.26	C-X-C chemokine receptor type 4/Endolysin chimeric protein
3oe6.1.A	30.63	homo-dimer	BLAST	X-ray	3.20Å	0.35	0.26	C-X-C chemokine receptor type 4, Lysozyme Chimera
3oe8.1.A	30.63	homo-dimer	BLAST	X-ray	3.10Å	0.35	0.26	C-X-C chemokine receptor type 4, Lysozyme Chimera
3odu.1.B	30.63	homo-dimer	BLAST	X-ray	2.50Å	0.35	0.26	C-X-C chemokine receptor type 4, Lysozyme Chimera
3odu.1.A	30.63	homo-dimer	BLAST	X-ray	2.50Å	0.35	0.26	C-X-C chemokine receptor type 4, Lysozyme Chimera
3oe8.1.B	30.63	homo-dimer	BLAST	X-ray	3.10Å	0.35	0.26	C-X-C chemokine receptor type 4, Lysozyme Chimera
3oe8.2.A	30.63	homo-trimer	BLAST	X-ray	3.10Å	0.35	0.26	C-X-C chemokine receptor type 4, Lysozyme Chimera
3oe0.1.A	30.63	hetero-oligome	BLAST	X-ray	2.90Å	0.35	0.26	C-X-C chemokine receptor type 4, Lysozyme Chimera
3oe9.1.B	30.63	homo-dimer	BLAST	X-ray	3.10Å	0.35	0.26	C-X-C chemokine receptor type 4, Lysozyme Chimera
3oe9.1.A	30.63	homo-dimer	BLAST	X-ray	3.10Å	0.35	0.26	C-X-C chemokine receptor type 4, Lysozyme Chimera
4rwd.1.A	31.82	monomer	BLAST	X-ray	2.70Å	0.36	0.26	Chimera protein of Human Delta-type opioid receptor and Escherichia coli Soluble cytochrome b562
4rwa.1.A	31.82	monomer	BLAST	X-ray	3.28Å	0.36	0.26	Chimera protein of Human Delta-type opioid receptor and Escherichia coli Soluble cytochrome b562
4qin.1.A	11.81	homo-dimer	HHblits	X-ray	2.60Å	0.24	0.3	Smoothened homolog/Soluble cytochrome b562 chimeric protein
4o9r.1.A	11.81	monomer	HHblits	X-ray	3.20Å	0.24	0.3	Smoothened homolog/Soluble cytochrome b562 chimeric protein
4qim.1.A	11.81	monomer	HHblits	X-ray	2.61Å	0.24	0.3	Smoothened homolog/Soluble cytochrome b562 chimeric protein
4zud.1.A	32.71	monomer	BLAST	X-ray	2.80Å	0.37	0.25	Chimera protein of Soluble cytochrome b562 and Type-1 angiotensin II receptor
4yay.1.A	32.71	monomer	BLAST	X-ray	2.90Å	0.37	0.25	Soluble cytochrome b562,Type-1 angiotensin II receptor
4xt3.1.A	33.98	hetero-oligome	BLAST	X-ray	3.80Å	0.36	0.25	G-protein coupled receptor homolog US28
4xt1.1.A	33.98	hetero-oligome	BLAST	X-ray	2.89Å	0.36	0.25	G-protein coupled receptor homolog US28

5cxv.1.A	32.38	monomer	BLAST	X-ray	2.70Å	0.34	0.25	Muscarinic acetylcholine receptor M1,Endolysin,Muscarinic acetylcholine receptor M1
4daj.1.A	32.67	monomer	BLAST	X-ray	3.40Å	0.36	0.24	Muscarinic acetylcholine receptor M3, Lysozyme
4daj.2.A	32.67	monomer	BLAST	X-ray	3.40Å	0.36	0.24	Muscarinic acetylcholine receptor M3, Lysozyme
4u14.1.A	32.67	monomer	BLAST	X-ray	3.57Å	0.36	0.24	Muscarinic acetylcholine receptor M3,Endolysin,Muscarinic acetylcholine receptor M3
4u16.2.A	32.67	monomer	BLAST	X-ray	3.70Å	0.36	0.24	Muscarinic acetylcholine receptor M3,Lysozyme,Muscarinic acetylcholine receptor M3
4u16.1.A	32.67	monomer	BLAST	X-ray	3.70Å	0.36	0.24	Muscarinic acetylcholine receptor M3,Lysozyme,Muscarinic acetylcholine receptor M3
4zj8.1.A	37	monomer	BLAST	X-ray	2.75Å	0.37	0.24	human OX1R fusion protein to P.abysii glycogen synthase
2ks9.1.A	29.79	hetero-oligome	BLAST	NMR	NA	0.36	0.22	Substance-P receptor
4s0v.1.A	38.04	monomer	BLAST	X-ray	2.50Å	0.37	0.22	Human Orexin receptor type 2 fusion protein to P. abysii Glycogen Synthase
5dhh.1.A	32.97	monomer	BLAST	X-ray	3.00Å	0.37	0.22	GPCR-BRIL Chimera
4ea3.1.A	32.97	homo-dimer	BLAST	X-ray	3.01Å	0.37	0.22	Fusion protein of Nociceptin receptor and cytochrome b562
5dhh.2.A	32.97	monomer	BLAST	X-ray	3.00Å	0.37	0.22	GPCR-BRIL Chimera
4k5y.3.A	11.96	monomer	HHblits	X-ray	2.98Å	0.26	0.22	Corticotropin-releasing factor receptor 1, T4-Lysozyme chimeric construct
4k5y.1.A	11.96	monomer	HHblits	X-ray	2.98Å	0.26	0.22	Corticotropin-releasing factor receptor 1, T4-Lysozyme chimeric construct
4k5y.2.A	11.96	monomer	HHblits	X-ray	2.98Å	0.26	0.22	Corticotropin-releasing factor receptor 1, T4-Lysozyme chimeric construct
2lot.1.A	30.77	monomer	HHblits	NMR	NA	0.33	0.09	Apelin receptor
2lou.1.A	30.77	monomer	HHblits	NMR	NA	0.33	0.09	Apelin receptor
2low.1.A	30.77	monomer	HHblits	NMR	NA	0.33	0.09	Apelin receptor
2lov.1.A	30.77	monomer	HHblits	NMR	NA	0.33	0.09	Apelin receptor
2koe.1.A	23.53	monomer	HHblits	NMR	NA	0.34	0.08	human cannabinoid receptor 1 - helix 7/8 peptide
1hof.1.A	31.03	monomer	HHblits	NMR	NA	0.34	0.07	ALPHA-2A ADRENERGIC RECEPTOR
1hll.1.A	31.03	monomer	HHblits	NMR	NA	0.34	0.07	ALPHA-2A ADRENERGIC RECEPTOR
1ho9.1.A	31.03	monomer	HHblits	NMR	NA	0.34	0.07	ALPHA-2A ADRENERGIC RECEPTOR
1hod.1.A	31.03	monomer	HHblits	NMR	NA	0.34	0.07	ALPHA-2A ADRENERGIC RECEPTOR
2ki9.1.A	28.57	monomer	HHblits	NMR	NA	0.34	0.07	Cannabinoid receptor 2
1fdf.1.A	26.09	monomer	HHblits	NMR	NA	0.35	0.05	RHODOPSIN
1eds.1.A	23.81	monomer	HHblits	NMR	NA	0.34	0.05	RHODOPSIN

SUPPLEMENTARY TABLE 4

Clinicopathological data of prostate cancer cases

Unique TMA	Age	Gleason
1	61	6
2	76	7
3	79	7
4	68	6
5	74	7
6	73	7
7	68	7
8	80	7
9	63	7
10	76	7
11	58	7
12	82	9
